# Increasing phenological asynchrony between spring green-up and arrival of migratory birds

**DOI:** 10.1038/s41598-017-02045-z

**Published:** 2017-05-15

**Authors:** Stephen J. Mayor, Robert P. Guralnick, Morgan W. Tingley, Javier Otegui, John C. Withey, Sarah C. Elmendorf, Margaret E. Andrew, Stefan Leyk, Ian S. Pearse, David C. Schneider

**Affiliations:** 1 Department of Ocean Sciences, Memorial University of Newfoundland, St. John’s, NL, A1C 5S7 Canada; 2 Department of Ecology and Evolutionary Biology, University of Colorado, Boulder, CO 80309 USA; 3 The National Ecological Observatory Network, Boulder, CO 80301 USA; 4 Florida Museum of Natural History, University of Florida, Gainesville, FL 32611 USA; 5 Department of Ecology and Evolutionary Biology, University of Connecticut, Storrs, CT 06269 USA; 6 Department of Biological Sciences, Florida International University, Miami, FL 33199 USA; 7 School of Veterinary and Life Sciences, Murdoch University, Murdoch, WA 6150 Australia; 8 Department of Geography, University of Colorado, Boulder, CO 80309 USA; 9 Illinois Natural History Survey, Champaign, IL 61820 USA

## Abstract

Consistent with a warming climate, birds are shifting the timing of their migrations, but it remains unclear to what extent these shifts have kept pace with the changing environment. Because bird migration is primarily cued by annually consistent physiological responses to photoperiod, but conditions at their breeding grounds depend on annually variable climate, bird arrival and climate-driven spring events would diverge. We combined satellite and citizen science data to estimate rates of change in phenological interval between spring green-up and migratory arrival for 48 breeding passerine species across North America. Both arrival and green-up changed over time, usually in the same direction (earlier or later). Although birds adjusted their arrival dates, 9 of 48 species did not keep pace with rapidly changing green-up and across all species the interval between arrival and green-up increased by over half a day per year. As green-up became earlier in the east, arrival of eastern breeding species increasingly lagged behind green-up, whereas in the west—where green-up typically became later—birds arrived increasingly earlier relative to green-up. Our results highlight that phenologies of species and trophic levels can shift at different rates, potentially leading to phenological mismatches with negative fitness consequences.

## Introduction

Understanding whether and how species are able to adjust and adapt to climate change has become one of the most urgent challenges facing ecology. Climate change is projected to drive hundreds of bird species to extinction and greatly reduce the ranges of others^1, 2^, and is already impacting species richness and composition^3^. Despite these impacts, in recent decades a majority of species examined have shifted the timing (phenology) of key ecological events, such as migration or reproduction^4^, consistent with expectations under climate change^5^. For instance, birds appear to be laying eggs earlier^6^, especially among populations experiencing greater increases in temperature^7^. Earlier arrival of migrants on breeding grounds has also been reported^8, 9^, mirroring advances in spring vegetation phenology^10^.

More important than whether birds are shifting their phenologies is whether these shifts adequately compensate for a changing climate and resulting shifts in avian food resources that drive fitness. Individuals and species might be able to adjust rapidly: phenotypically plastic behavioural responses can track environmental conditions closely^11^, and even evolutionary changes in migratory behaviour which are generally expected to be much slower can occur rapidly^12^. Migratory birds, given their ability to rapidly move long distances, might appear to be among the most adaptable animals to climate change. Migration itself is partly an adaptation to intra-annual changes in climate, so additional inter-annual climatic changes might seem not to pose a problem for further adaptation. However, onset of long-distance migration in birds is primarily cued by physiological responses to photoperiod—which is annually consistent—yet conditions at their breeding grounds depend on climate—which is annually variable^13, 14^. To maximize fitness, birds must time their breeding phenology (including arrival on breeding grounds, breeding, egg laying, and fledging) to coincide with optimal habitat conditions and food availability. This means there is evolutionary incentive to correctly anticipate breeding site conditions while birds are still at their winter grounds, often thousands of kilometers distant. As climate at the breeding grounds changes, birds may be unable to adjust wintering ground departure times and transit speeds sufficiently to match their arrival with altered breeding resource phenology, particularly leaf growth and the closely associated emergence of herbivorous insects.

Arriving too early at breeding grounds can bring risk of freezing (due to cold temperatures) and hatching chicks before peak resource abundance, whereas arriving too late can mean fewer nest sites, fewer mates with successfully guarded territories, and declining resource abundance^15, 16^. As such, the loss of synchrony between insect emergence and migrant bird arrival phenology^17, 18^ can be accompanied by negative fitness consequences including reduced reproductive output and juvenile survival^19, 20^. The decoupling of the phenology across trophic levels can ultimately lead to population declines and biodiversity loss^20, 21^. Beyond impacts on birds, phenological asynchrony between birds and their insect prey can generate novel trophic cascades: for instance, a lack of predation on insects can cause insect outbreaks and subsequently increase defoliation of trees^22^.

We ask whether bird migration phenology has kept pace with the phenology of their food. To address this question, we undertook the first continental scale study of phenological asynchrony, presenting trends in the intervals between vegetation green-up and the arrival of 48 passerine bird species in North America for the period 2001–2012. Although only 12 years of data, the rapid pace of warming (approximately 0.024 °C per year in the contiguous U.S.^23^), a key driver of green-up, makes this is a sufficient period over which to test for substantial trends in phenological interval. We used mean green-up date as derived from satellite imagery as a proxy for timing of peak resource availability and as a yardstick with which to compare avian arrival^24, 25^. Green-up provides a reasonable proxy for passerine food availability—and a closer one than climatic conditions—because green-up timing is strongly correlated with the emergence of young insects that fuel migrants and form the primary food source for nestling passerine birds^25–29^. Green-up is also known to be a strong predictor of migratory and breeding phenology in birds^30, 31^.

We then compared green-up dates with migratory bird arrival dates, as estimated from continent-wide citizen science observations provided by eBird. The continental extent of this study complements local scale mechanistic investigations (e.g. refs 32 and 33) and enabled estimation of trends in phenological intervals throughout breeding ranges and across widely varying ecological conditions. We expected that phenological intervals would increase with increasingly earlier green-up, indicating a lack of adaptation to changing vegetation phenology.

## Results

### Species trends

**Figure 1:**
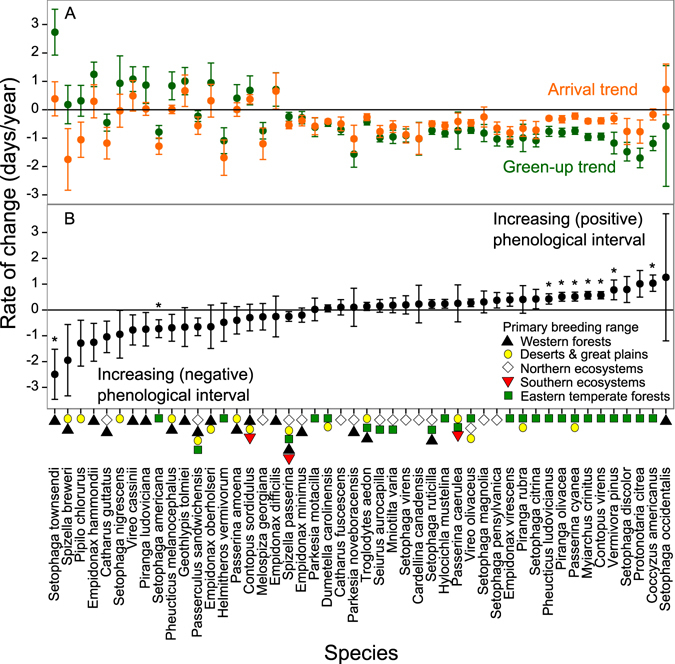
Trends in green-up date, bird arrival date, and phenological interval by species. Bars indicate +/− 1 standard error. Species are ranked by trend in phenological interval. In (**A**), positive values indicate arrival (orange) or green-up (green) are getting later, negative values indicate they are getting earlier. Note that for species on the right (with increasing phenological interval), green-up is getting earlier at a greater rate than is arrival. In (**B**), positive values indicate increasing phenological interval (black) between green-up and arrival date. Coloured symbols below the panel indicate the general location(s) of the species’ breeding range in North America. Asterisks indicate statistically significant trends in phenological interval (p < 0.05). Note that species on the right (with increasing positive phenological interval) tend to breed in Eastern Temperate Forests, whereas species on the left (with increasing negative phenological interval) tend to breed in Western Forests.

Over the 12 year period, green-up dates advanced significantly in 27 species’ breeding ranges, by a mean of 0.952 days/year, and became later in four species breeding ranges by a mean of 1.52 days/year (Fig. 1, Supplementary Table S1). Across all species, the mean trend in green-up date in cells making up a species’ breeding range (the mean of trends in Supplementary Table S1), was an advance of 0.372 ± 0.387 days/year. Similarly, arrival dates advanced significantly for 25 of 48 species (52%) by a mean of 0.669 days/year, while arrival became later only for one species, *Contopus sordidulus*, by 0.370 days/year (Fig. 1, Supplementary Table S2). Across all species, mean arrival advanced by 0.426 ± 0.340 days/year (mean of slopes in Supplementary Table S2). Frequently, when green-up changed significantly in a species’ range, a species’ arrival also changed significantly, and usually in the same direction; when both green-up and arrival changed significantly for a given species, they always became earlier.

Phenological intervals (date_arrival_ – date_green-up_) between green-up and arrival increased significantly in nine species (Fig. 1, Supplementary Table S3). We observed two types of increasing phenological interval: increasing positive intervals occurred when green-up increasingly preceded arrival, while increasing negative interval occurred when green-up became increasingly later relative to arrival over time. For species with increasing positive intervals, both green-up and arrival tended to become earlier over time. However, green-up advanced at a greater pace than did arrival. For the seven species with significantly increasing positive intervals, the intervals increased by mean = 0.630 days/year on average (Supplementary Table S3). For species with increasing negative intervals (mean = 1.60 days/year), green-up tended to get later over time but arrival trend was more variable, advancing for some species and getting later for others (Supplementary Table S3). For the latter group, the pace of change in arrival was typically slower than for green-up. Thus, for both species with increasing positive and negative intervals, greater phenological change was generally observed in green-up than in arrival. Still, we did not detect statistically significantly changing intervals for 39 of 48 species (81%) over this 12 year period (Fig. 1, Supplementary Table S3). Also, the number of species for which trends in phenological interval were considered significant differed with statistical adjustments for multiple comparisons (Supplementary Table S4), but significant effects remained with each approach. Because the phenological interval could increase positively or negatively, mean trend in phenological interval was only an advance of 0.092 days/year across all species, but the mean absolute effect size (change in any direction) across species was an increase in phenological interval of 0.575 ± 0.512 days/year (Supplementary Table S3).

**Figure 2:**
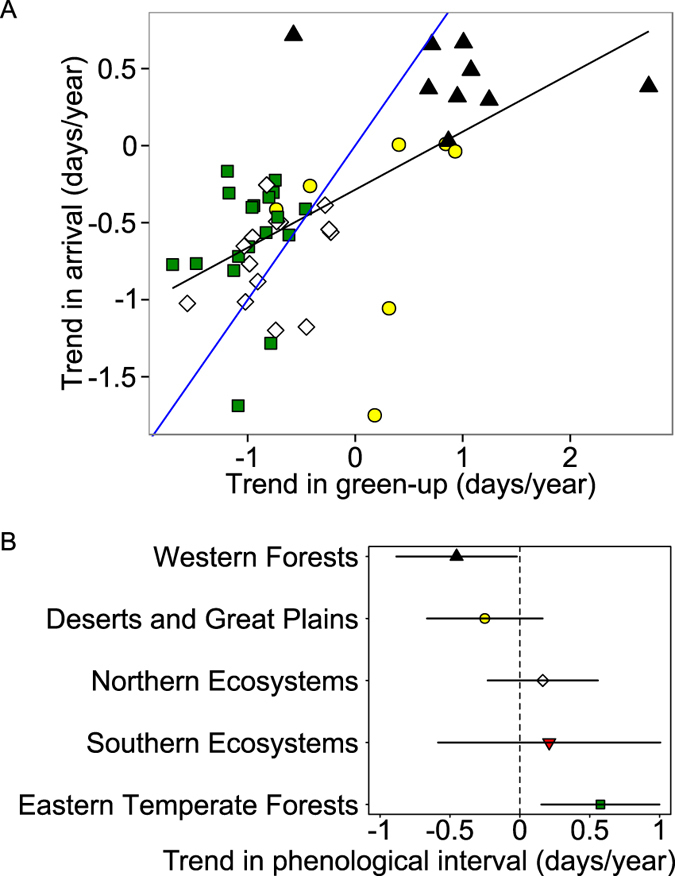
Ecoregional differences in arrival trend vs. green-up trend relationship and in explaining trend in phenological interval. (**A**) Relationship between trend in bird species arrival and trend in green-up. Each symbol represents a species, and symbol colour and shape indicate the primary ecoregion of the species’ breeding range, as defined in Fig. 1 (only the most occupied region shown). Black line indicates linear regression for all species (*p* < 0.001), blue line indicates where trend in arrival equals trend in green-up. The trend for all species was statistically significantly (*p* = 0.004) different from slope = 1 (blue line), indicating trend in arrival changed less than trend in green-up. (**B**) Ecoregions as linear regression coefficients predicting trend in phenological interval across species. Bars indicate 95% confidence intervals.

Across the majority of species where phenological intervals were increasing, the advance in arrival date lagged behind the advance in green-up date (Fig. 1). Similarly, a linear regression between arrival trend and green-up trend across species (Arrival trend = 0.376 × Green-up trend −0.286, *adj. R* ^2^ = 0.350, *p* < 0.001) showed that for species where green-up was getting earlier, bird arrival was also getting earlier. However, that slope was significantly (*p* < 0.004) different from 1. This indicates that arrival was not getting earlier as quickly as was green-up (Fig. 2a).

### Geographic trends

Trends in phenological intervals differed by region (Figs 1 and 2b, Supplementary Fig. S2). We partitioned North America into five large ecoregions for which we expected distinct patterns in vegetation phenology and possible differences in migration phenology of birds, but for which we had no *a priori* expectation for trends in phenological intervals (Supplementary Fig. S3). Species that breed primarily in Eastern Temperate Forests tended to display increasing positive phenological interval over time, whereas species breeding in the Western Forests tended to exhibit increasing negative trends in phenological interval (Fig. 2b). Species that breed primarily in the Deserts and Great Plains, and Northern and Southern Ecosystems tended to exhibit little change in phenological interval (Fig. 2b).

**Figure 3:**
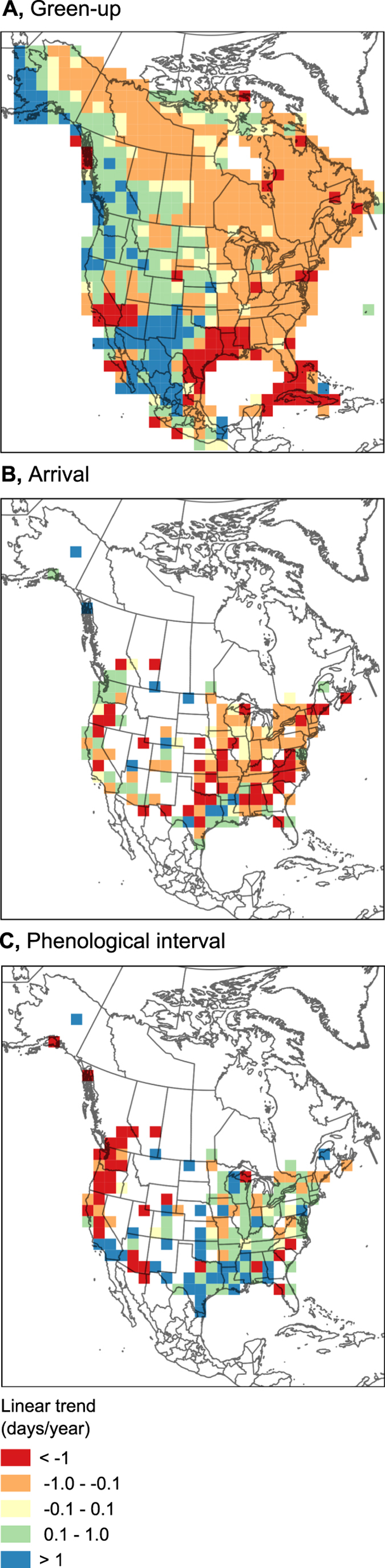
Maps of linear mixed model slopes in (**A**) green-up, (**B**) bird arrival, and (**C**) phenological interval between green-up and arrival for the period 2001–2012. Warmer colours indicate green-up (**A**) or arrival (**B**) getting earlier, or increasing negative phenological interval (**C**). Cooler colours indicate green-up or arrival getting later, or increasing positive phenological interval. Slopes for b and c are slopes across all species. Maps created with QGIS 2.6, www.qgis.org.

These regionally varying trends in phenological interval were mirrored by continental-scale variation in underlying green-up and arrival patterns. From 2001–2012, green-up advanced throughout eastern and northern North America, but unexpectedly lagged in much of western North America (Fig. 3a). We expected arrival trends would follow green-up trends. Arrival of migratory birds occurred earlier in most areas, especially in eastern North America (Fig. 3b), consistent with previous work^9^. In some areas however, birds arrived later, notably around the Gulf of Mexico and the Pacific Northwest. While the delayed arrival in the west is consistent with delayed green-up in the west, arrival trends around the Gulf of Mexico became later while green-up became earlier, an incongruity suggesting that bird arrival times to these areas were determined by other factors.

## Discussion

Mismatches in cross-trophic species interactions can impact demography under climatic change^34, 35^, potentially threatening bird populations if these mismatches are too great or increase too rapidly. Population dynamics depend on minimizing phenological mismatch of consumers and peak resource availability^36, 37^. For migratory birds, arrival to the breeding grounds is a critical phenological event that sets the stage for the remainder of the breeding season, impacting offspring survival and performance^38^. While the optimal date of arrival is likely a function of conditions at the breeding grounds, actual arrival date can be influenced by conditions at the wintering grounds and along the migratory route^39–41^. Still, migratory timing can be very tightly linked to conditions at the breeding grounds, with the timing of spring arrival driving phenological events throughout the annual cycle^42^.

Although birds have had to adapt to climatic shifts and resulting asynchronies with resources throughout their evolutionary history, the current rate and magnitude of change have exceeded normal bounds^43^, raising the question of whether migrant bird populations have been able to keep pace with key phenological events. We found that a majority of migratory bird species adjusted the date of their arrival, usually in the direction (earlier or later) that vegetation green-up changed. Thus, it appears the phenology of migratory arrival is more often than not responding to climate change. In a similar set of North America passerine migrants, median capture date advanced approximately 1 day/degree °C over 40 years, but spring vegetation phenology advanced at 3.2 days/degree °C^39^. Here, we found that the interval between bird arrival and vegetation green-up increased significantly in nine species of the 48 examined. These increases in phenological intervals were detected despite only 12 years of data, and may become more apparent in more species over longer time series. Across all species, the mean growth in the phenological interval between arrival and green-up was 0.575 days/year. Thus, although populations adjusted their arrival, they did not do so sufficiently to keep pace with changing green-up.

In Europe, migratory pied flycatchers (*Ficedula hypoleuca*)^32^ and collared flycatchers^44^ advanced egg laying dates, but not as much as necessary to coincide with peak insect abundance, while in the eastern United States, Marra *et al*.^39^ found that median capture dates of 42 species of long-distance passage migrants advanced, but lagged behind advances in plant budburst. In one contrasting study, Cresswell and McCleery^45^ determined that non-migratory great tits (*Parus major*) in England advanced their laying phenology earlier than the phenology of their insect prey, but others have found stable or increasing phenological intervals in this species^27, 44^. Our results are generally comparable to previous studies of individual species’ responses to food availability at individual study sites which have benefitted from longer time series generally dating to the 1960s or 1970s. While we observed an advance in green-up of 0.372 days/year across the breeding ranges of 48 bird species, advances in caterpillars have ranged from 0.189 to 0.87 days per year in (European) studies reviewed by Visser *et al*.^27^. Similarly, we observed that mean arrival advanced by 0.426 days/year across species, whereas studies of birds responding to caterpillars reviewed by Visser *et al*. (4 species, 11 sites) showed advances ranging from 0.17 to 0.52 days/year. Despite these similarities, our observations of trends in green-up (1.699 days/year earlier to 2.730 days/year later) and arrival ranged more widely (1.750 days/year earlier to 0.716 days/year later) than previous work, suggesting increased variation in phenological trends across species and their breeding ranges. Our results for North American passerines generally show a greater magnitude of increase in phenological interval, at 0.575 days/year, than reported in Visser *et al*.^27^, but it is unclear if this difference is due to different continents, species, length of time series, spatial scope, recent acceleration of growth in phenological interval, or other factors. Overall, birds appear to be exhibiting phenotypic plasticity in response to their changing environmental conditions, but the pace and magnitude of phenological change in vegetation may well exceed the extent of this plasticity.

Trends in phenological interval varied by ecoregion, an unanticipated result. We found that although many eastern bird species have advanced their arrival times, this advance lagged behind increasingly early green-up and as a result, the interval between arrival and green-up has increased over the 2001 to 2012 period. In a similar yet unexpected process, species of the Western Forests experienced increasingly later green-up, but did not arrive late enough to match those changing conditions. These species also face increasing phenological intervals despite the direction of phenological shift. To our knowledge, this is the first demonstration of strong regional variation in phenological interval trends in migratory birds. Regional variation in trends in arrival dates (but not intervals) has, however, been reported for European and Australian birds^8, 46^. The effects of recent climate change on the phenology of migratory birds, therefore, are strongly dependent on region. Conclusions drawn for one biome or region, or from a single species, should be very cautiously applied at larger geographic scales or to entire avian communities.

Ecoregional differences in trends in arrival dates and phenological intervals may be the result of birds from different ecoregions tending to have differing migration distances and origins (wintering grounds). For instance, trends in arrival dates can depend on migration distance^47, 48^. However, our results, which show that the geography of green-up trends strongly explains trends in phenological intervals, may suggest a more limited role for species traits such as migration distance, in explaining those trends. Nonetheless, further examining the role of dispersal-related species traits, especially when dissecting finer-scale aspects of species tracking, is a ripe area for future research.

Our study provides an important link between mechanistic ecological studies at local scales and broader changes in the climate at continental scales. Our work benefitted from continental-scale data sets with which the phenologies of birds and vegetation could be united. However, such broad-scale data sets often lack the direct mechanistic linkages that are gained from many local data sets. For example, green-up is not a direct measure of food availability, which has strong mechanistic linkages to arrival phenology. However, we view green-up as a strong index point for arrival timing of migratory insectivorous birds^24, 25^ for the following reasons. First, green-up predicts the increase in availability of insects as bird resources. Most foliage gleaning birds consume primarily herbivorous insects^49, 50^, whose biomass in turn increases as a direct response to green-up^26, 27, 51^. Second, green-up occurs at comparable temperature thresholds to the flight of many insects^52^ and degree-day models predict both leafing phenology of plants and flight of insects^53^. Third, birds incur costs for later arrival. While it has not yet been established whether edible arthropod biomass generally decreases at times beyond early spring^52^, anti-herbivore allelopathic chemicals tend to increase throughout the growing season^54^ and birds may face additional costs with later arrival such as fewer available nest sites and fewer available mates with territories^15, 16^.

Despite these biological linkages between phenologies of birds and green-up, we note that the interval between green-up and bird arrival is not expected to be zero (only that the interval should be consistent under stable interannual conditions). Ideally, phenologies of all forage resource groups would be combined with detailed phenologies of bird species’ reproductive events, including territory establishment, egg laying, hatching, and fledging. Lacking such data at broad scales, we suggest that answering the question of phenological mismatch across trophic levels will require a dual approach in which direct observation and experimentation at local scales tests causal mechanisms, while spatially broad datasets are employed to scale up to the continental level and enable regional and cross-species comparisons.

Two methodological notes are worth mentioning. In this study, we have considered the sensitivity of observed trends in phenological interval to our estimation of arrival date as the inflection point of a logistic model fit to the proportion of presences to absences over time. However, modeling approaches for phenological data are an area of current debate. For instance, Newson *et al*.^55^ employed generalized additive models to characterize multiple phenological events over the year, and approaches such as this that employ non-parametric smoothing allow maximum model shape flexibility. Linden *et al*.^56^ empirically evaluated parametric and nonparametric models and suggested that parametric models are most often preferable for modeling the arrival period. Regardless of the approach, however, the sensitivity of phenological interval to arrival date estimation should be evaluated, because the estimation could be influenced by data following the arrival period. In the Methods section and in Supplementary Note 1 we detail the sensitivity of our results to arrival estimation including impacts of passing (nonbreeding) migrants, latitudinal effects, estimation window size, and proportional position of the arrival estimation within the arrival period.

Additionally, our analyses leveraged observations from eBird; citizen-science data varies in quality and is spatially and temporally heterogeneous, and eBird’s citizen-science data is not immune to this limitation^57–59^. However, eBird’s data remains the richest source of presence-absence data for birds and can offer insights at previously unexplored spatial scopes, as well as across many species concurrently^9, 60^. Our confidence in eBird data as appropriate for estimating mean arrival dates of bird populations is supported by the following: i) the law of large numbers, suggesting that richer data may be less susceptible to outliers, misidentifications, or observer biases unless these vary systematically; ii) our demonstration that the number of checklists submitted per observer, which can be related to the number of species reported, does not vary systematically by region (Supplementary Note S2); iii) our filtering of arrival date estimates to those species-cell-years with good model fits; iv) thorough model sensitivity analyses provided in Supplementary Note S1; v) the previous use of this method and data by Hurlbert *et al*.^9^; vi) similar limitations in citizen science data as professionally collected data^58, 59^; vii) similar accuracy and data quality in citizen science data as professionally collected data^59^; viii) general conformity of results derived from eBird data with experimental results, while offering novel insights^60^. Methods for further refinement of models to account for potential biases are actively being developed^58, 59^.

Although we observed migrant North American birds failing to keep pace with the rapidly shifting phenology of vegetation, the specific demographic and ecosystem consequences of these trends remain unknown. Demographic trends are predicted by trends in climatic suitability^61^, and if birds are unable to keep pace with their changing environment, reduced fitness for individuals^27, 33^, reduced population sizes^48, 62^, and in the extreme, extirpations, could result. Declines in migratory bird populations attributable to climate-related phenological mismatch have already been observed^21, 62–65^, and phenological synchrony can be comparable to food availability and conspecific density in explaining reproductive success^66^, but the sensitivity of different demographic parameters to mismatch are still poorly understood. For instance, density dependent compensation may buffer against mismatch to maintain populations^67^ but selection favouring reduced phenological interval can be relaxed when populations have declined^68^. We expect demographic response rates to differ across species (according to their traits) and regions, highlighting the importance of both regional scale analytical approaches and of continental-scale programs for monitoring the occurrence and demography of sensitive widespread taxa such as birds. Whatever the demographic consequences of phenological asynchrony may be, it is clear that even over the relatively short time span of 12 years, this mismatch is increasing for a large number of migratory bird species, providing evidence that trophic interactions are failing to keep pace with a rapidly changing climate.

## Methods

We divided North America into a grid of 200 km × 200 km ‘cells’ based on the North America Albers Equal Area Conic Projection (NAD83). This resolution was selected to be sufficiently coarse to permit arrival to be estimated in a robust way from the data available from citizen science efforts, yet fine enough to allow meaningful analyses using these cells as spatial (analytical) units.

We estimated bird arrival dates using data from eBird (www.ebird.org, “basic” dataset, accessed May 7, 2014), a database of citizen science checklists, following Hurlbert & Liang (2012). Although detection probability of birds in this database likely varies with observer ability, species traits, and temporal and spatial extent of observations, we assumed that due to the large number of observers, variation in the composite data did not suffer from seasonal, annual, or geographic biases in estimates of arrival date. We selected 48 passerine species for analysis based on the following criteria: i) common species with large breeding ranges; ii) breeding range primarily located near populated areas of North America and thus likely to be well-sampled via citizen science endeavors; and iii) breeding range largely non-overlapping with winter range. To estimate arrival dates, we first masked eBird records to each species’ North American breeding range, which were obtained from NatureServe (http://services.natureserve.org). Second, for all records located in a grid cell for a given year, we used R (version 3.1, R Foundation for Statistical Computing, Vienna, Austria) to fit a logistic model to the dates of presences (species observed) and assumed absences (species not observed on checklist where all species present were considered reported) between Julian days 80 to 180, with the proportion of presences as the response variable (Supplementary Fig. S1). The day 80 to 180 window we used, though arbitrary, was selected to encompass the probable mean arrival dates. Our logistic models allowed for (maximum) asymptotes <1, since the proportion of surveys positively reporting a given species rarely approached 1. We used the inflection point of the fitted logistic model as the estimated mean arrival date of a given species to a given cell in a given year. We repeated this estimation procedure for all species in each cell and year. Where data were sparse (<10 presences per cell-year), logistic models could not be reliably fit to the data so these grid cells were excluded from analysis. To limit potential biases generated in instances where the logistic models poorly fit bird observation data over time, we further excluded all arrival date estimates from models with poor fit as indicated by the residual deviance divided by residual degrees of freedom was >4, and all models with low statistical significance (*p* < 0.05). The estimated mean arrival date was utilized rather than first arrival date or the range in arrival dates because it is less sensitive to outliers and more accurately represents the arrival of the population^9^. Although arrival dates vary in their uncertainty, we did not weight mean arrival date estimates by the inverse variance because this variation represents not only uncertainty in the arrival estimate but also the variability in when individuals of a species arrive (for instance, whether a population arrives more synchronized versus variably over time).

We estimated green-up dates using data from MODIS product MCD12Q2 Land Cover Dynamics V005^69^. It provides the date of “onset of greenness increase” which is derived from fitting a logistic model to Enhanced Vegetation Index (EVI) data, for each year, computed from a Nadir Bidirectional Reflectance Distribution Function-Adjusted Reflectance function for an 8 day period at a 500 m pixel resolution^70, 71^. We aggregated the dates in each 200 km grid cell using QGIS (version 2.6, Open Source Geospatial Foundation Project, Boston, USA, www.qgis.org) and its “zonal statistics” function to determine the mean date of onset of greenness each year across all MODIS pixels and treated this as the estimated date of green-up. There is generally high correspondence of satellite-derived phenological data and field observations of vegetation^72, 73^.

We calculated phenological interval as the difference between arrival and green-up dates for a given species, in a given cell and year. The interval is typically thus a positive number, as birds typically arrive following green-up. However, when birds arrive prior to green-up, phenological interval is a negative number. Since green-up is an index of phenology of food resources but is several mechanistic steps removed from optimal bird arrival dates (which are likely species-specific), we refrain from placing any functional value (positive or negative) on mean interval for each species. Rather, the interval as measured here is an index of phenology, and if birds are synchronized with respect to local phenology, we expect the measured interval to be constant over time, even with year-to-year variation. Linear trends in phenological interval over time, however, indicate that bird species are increasingly not synchronized with vegetation. We do not presume that the phenological interval between green-up and arrival ought to be zero (that green-up and arrival should be synchronous) to maximize fitness, or that arrival strictly determines other phenological events in birds.

To explore variation in trends of green-up, arrival and phenological interval across species (as in Fig. l), we determined (for each of those responses) slopes for each species by fitting linear mixed models, with ‘grid cell’ as a factor with random intercept, to estimated values across the years 2001 to 2012. Similarly, to explore variation in trends of green-up, arrival and phenological interval across space (as in Fig. 3), we determined slopes in green-up, arrival and phenological interval at each cell by fitting linear mixed models, with ‘species’ as a factor with random intercept, to estimated values across the 2001 to 2012 period. Figure 3 shows these slopes mapped across cells. These and all other maps used the North America Albers Equal Area Conic projection and were created with QGIS (version 2.6, Open Source Geospatial Foundation Project, Boston, USA, www.qgis.org) and display U.S. Geological Survey North American political boundaries (2006 version).

We defined increasing positive phenological interval as a positive trend in the difference between arrival and green-up, when arrival increasingly lags green-up. We defined increasing negative phenological interval as the negative trend in the difference between arrival and green-up, when arrival increasingly precedes green-up.

To investigate broader geographic trends, we defined five large ecoregions in North America (Supplementary Fig. S2) by combining similar Level 1 ecological regions from the Commission for Environmental Cooperation (http://www.epa.gov/wed/pages/ecoregions.htm). We then assigned species to an ecoregion if either their breeding range covered >33% of an ecoregion, and/or if an ecoregion covered >33% of the species’ range (Supplementary Fig. S3).

To test if arrival changed to the same degree as green-up across species, we performed a linear regression of trend in arrival by trend in green-up, with species as the observational unit, and where ‘trend’ indicates the mean slope of mixed model with cell as a factor with random intercept as calculated above. To test for ecoregions in explaining trend in phenological interval, we performed a multiple linear regression with ecoregions as predictors of trend in phenological interval and plotted the regression coefficients with 95% confidence intervals.

We assumed that the logistic curve was an appropriately shaped model of how occupancy varies over time as bird populations arrive to a cell. Although nonparametric^55^ and parametric^9^ models are both employed in phenological studies of birds, parametric models are often preferable^56^. Still, the logistic might not be appropriate if a large proportion of migrants arriving to a cell pass through, rather than remain in the cell. In this case occupancy would be expected to peak and decline rather than reach an asymptote, thereby resulting in poor model fits particularly in the post-arrival period. Additionally, if birds are less sensitive to environmental conditions when passing through a cell than when settling to breed, synchrony may be less important for their fitness. Although we were unable to differentiate among those birds passing through a cell and those remaining to breed, we excluded arrival estimates from logistic models with poor fits or that lacked statistical significance (discussed above). Furthermore, we tested the sensitivity of our results to passing migrants in three ways (see Supplemental Note 1). First, because passing birds are expected to decrease with latitude, we re-estimated trends in phenological interval while controlling for latitude and found that more species showed significant trends in interval, suggesting our estimates of the number of species with significant trends in phenological interval was conservative. Second, if passing migrants impact occupancy over time then arrival date estimation may be sensitive to the range of dates over which arrival is estimated (‘window size’), and this effect would vary with latitude^9^. We found that the sensitivity of arrival to window size did not vary with latitude. Third, we re-estimated arrival dates based on alternative points on the logistic curves of proportion of presences over time, focusing especially on earlier arrival given that there may be fitness benefits to early arrival. We found that with earlier arrival estimates more species showed significant trends in phenological interval and that the mean trend in phenological interval was stronger, suggesting our estimates may have been conservative. A full discussion of these secondary analyses of arrival estimation sensitivities and their rationales is provided in Supplementary Note S1.

## Electronic supplementary material

Supplementory Information
