# Supplementary material for: Increasing phenological asynchrony between spring green-up and arrival of migratory birds
Source: Sci Rep. 2017 May 15;7:1902. doi: 10.1038/s41598-017-02045-z (PMC5432526; doi:10.1038/s41598-017-02045-z)
Supplement: Supplementary file 1 — Supplementory Information [file 41598_2017_2045_MOESM1_ESM.pdf]

Supplementary Information

This file provides supplementary information to:

Mayor, Stephen J., Robert P. Guralnick, Morgan W. Tingley, Javier Otegui, John C. Withey, Sarah C. Elmendorf, Margaret E. Andrew, Stefan Leyk, Ian S. Pearse, and David C. Schneider. 2017. Increasing phenological asynchrony between spring green-up and arrival of migratory birds. Scientific Reports.

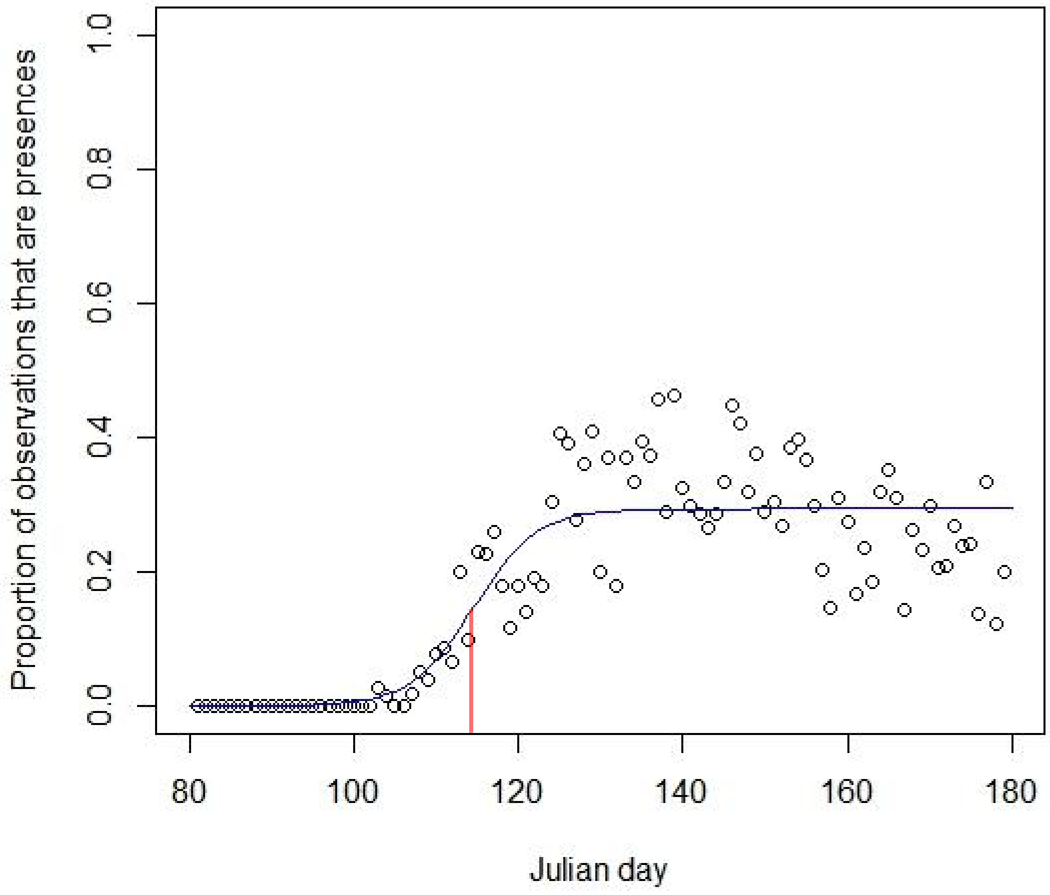

Supplementary Figure S1. Method by which arrival date was estimated from eBrid records. Method demonstrated for *Troglodytes aedon* in 2008, at a single cell. For each given Julian day between 80 and 180, the proportion of eBird checklists that were presences was determined (dots). Next, a logistic model (blue curve) was fit to those proportions. Logistic models allowed for asymptotes < 1, since the proportion of surveys positively reporting a given species rarely approached 1. Finally, the inflection point of the logistic (red line) was taken as the estimated arrival date. Note that this estimate is later than the sometimes used "first arrival dates", and can be considered a population level mean arrival date.

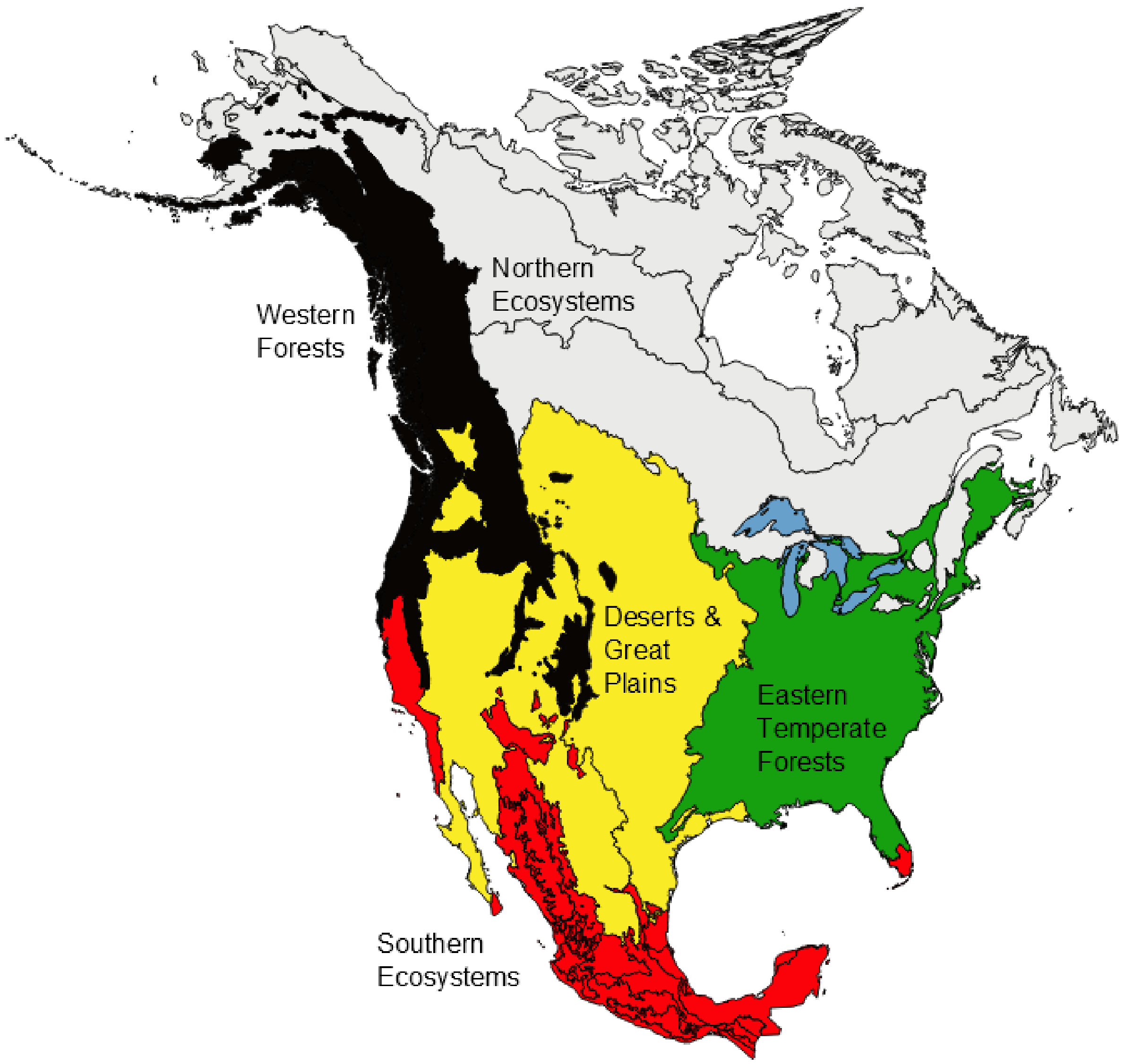

Supplementary Figure S2. Map of ecoregions. Ecoregions were derived by combining similar and geographically adjacent Level 1 ecological regions from the Commission for Environmental Cooperation. (Commission for Environmental Cooperation Working Group. 1997. Ecological regions of North America - toward a common perspective. Montréal, Canada). Black lines within an ecoregion indicate Level 1 ecological region boundaries for comparison. As an example, “North American Deserts” and “Great Plains” Level 1 ecological regions were combined into the “Deserts and Great Plains” ecoregion used in this study. Map created with QGIS 2.6, www.qgis.org.

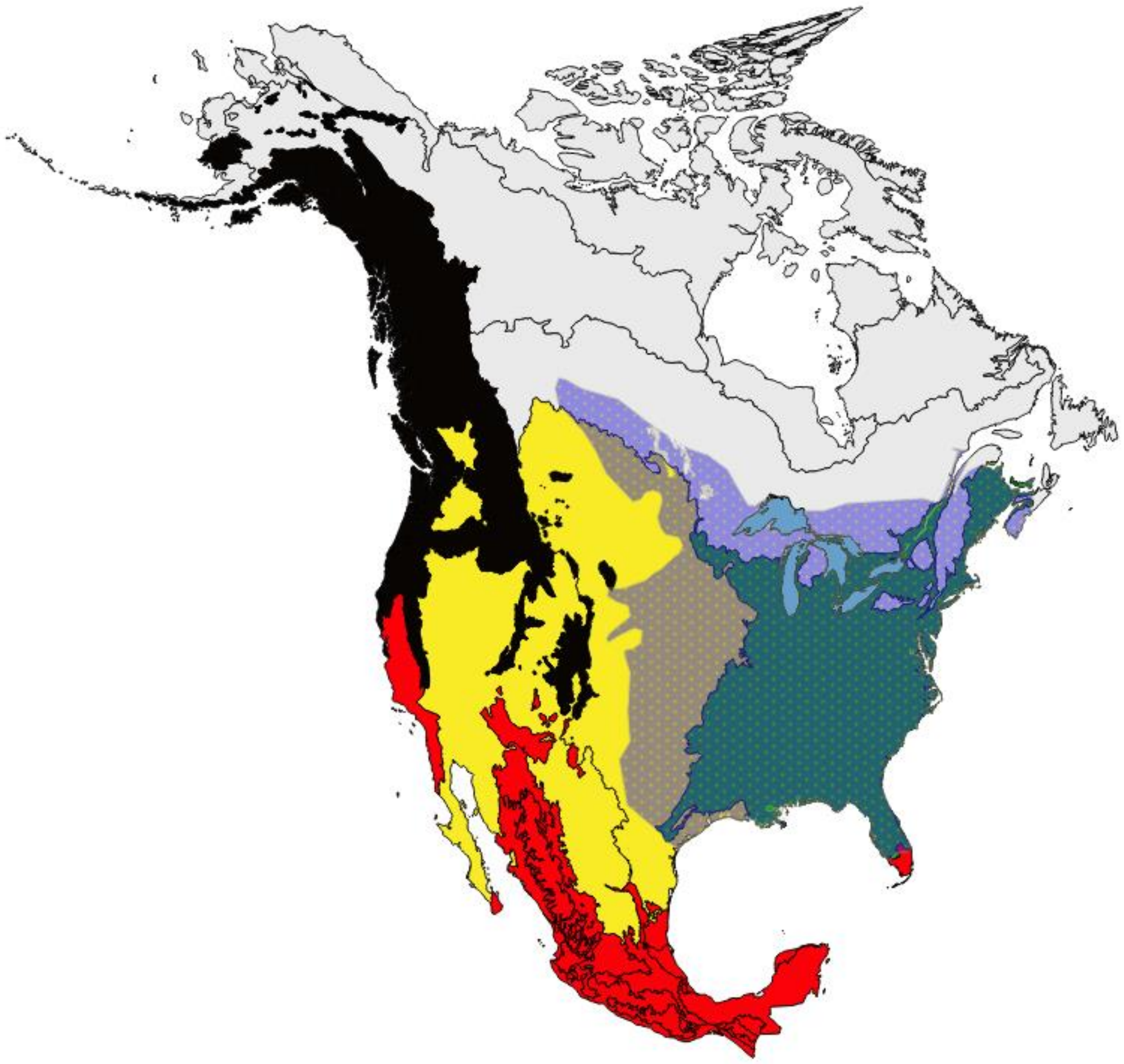

Supplementary Figure S3. Method by which ecoregions were assigned to species. Ecoregions as in Supplementary Fig. S1. First, breeding range of *Myiarchus crinitus*, in blue and dotted, overlaid on map of ecoregions. Second, an ecoregion was assigned to the species if > 33 % of range overlapped with an ecoregion, and/or if > 33 % of ecoregion overlapped with species range. In this case, near complete overlap with Eastern Temperate Forests resulted in assignment of that ecoregion to the species, but insufficient overlap with Northern Ecosystems or Deserts and Great Plains resulted in no assignment of those ecoregions to the species. Map created with QGIS 2.6, www.qgis.org.

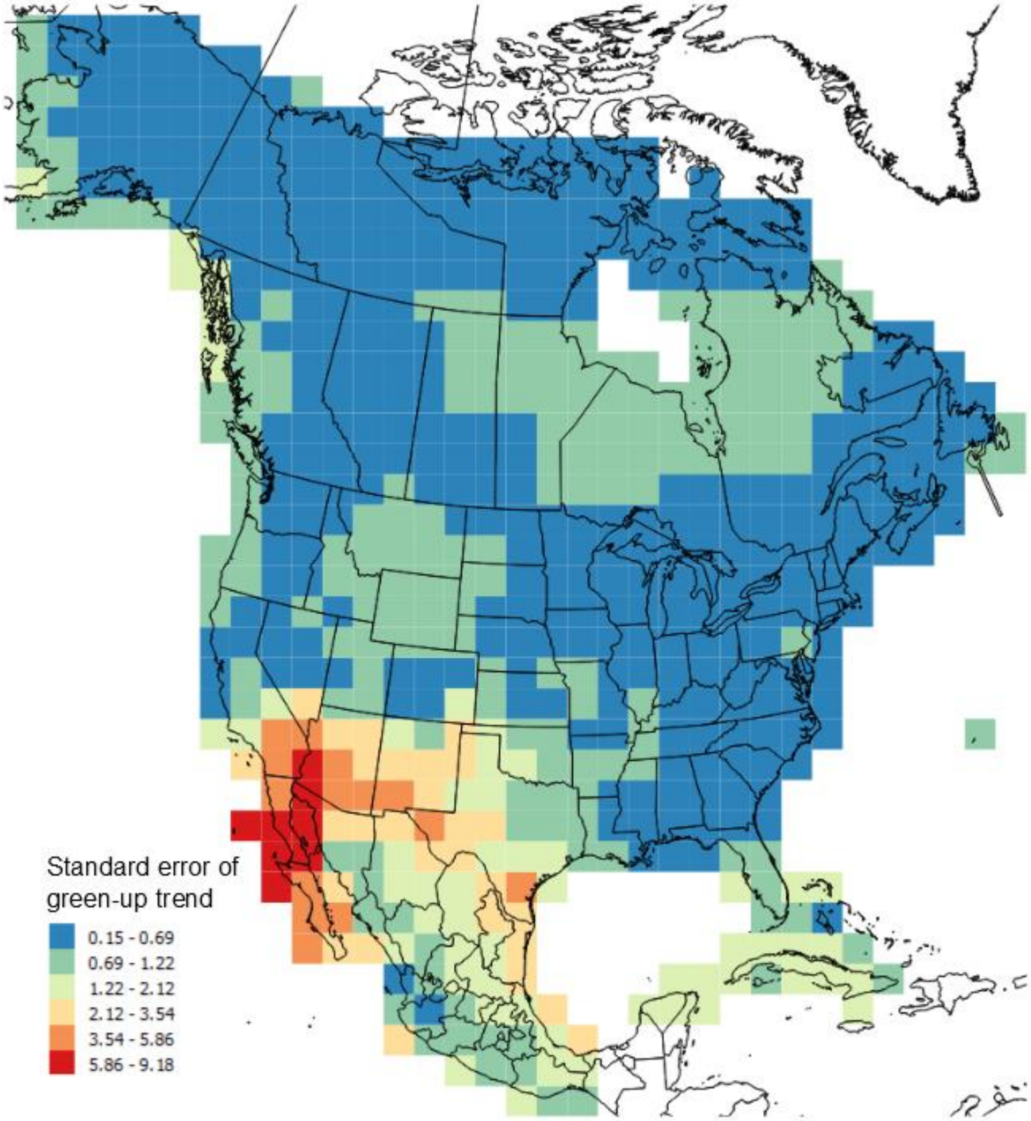

Supplementary Figure S4. Standard error of trend in green-up. Warmer colours indicate higher standard error. Map created with QGIS 2.6, www.qgis.org.

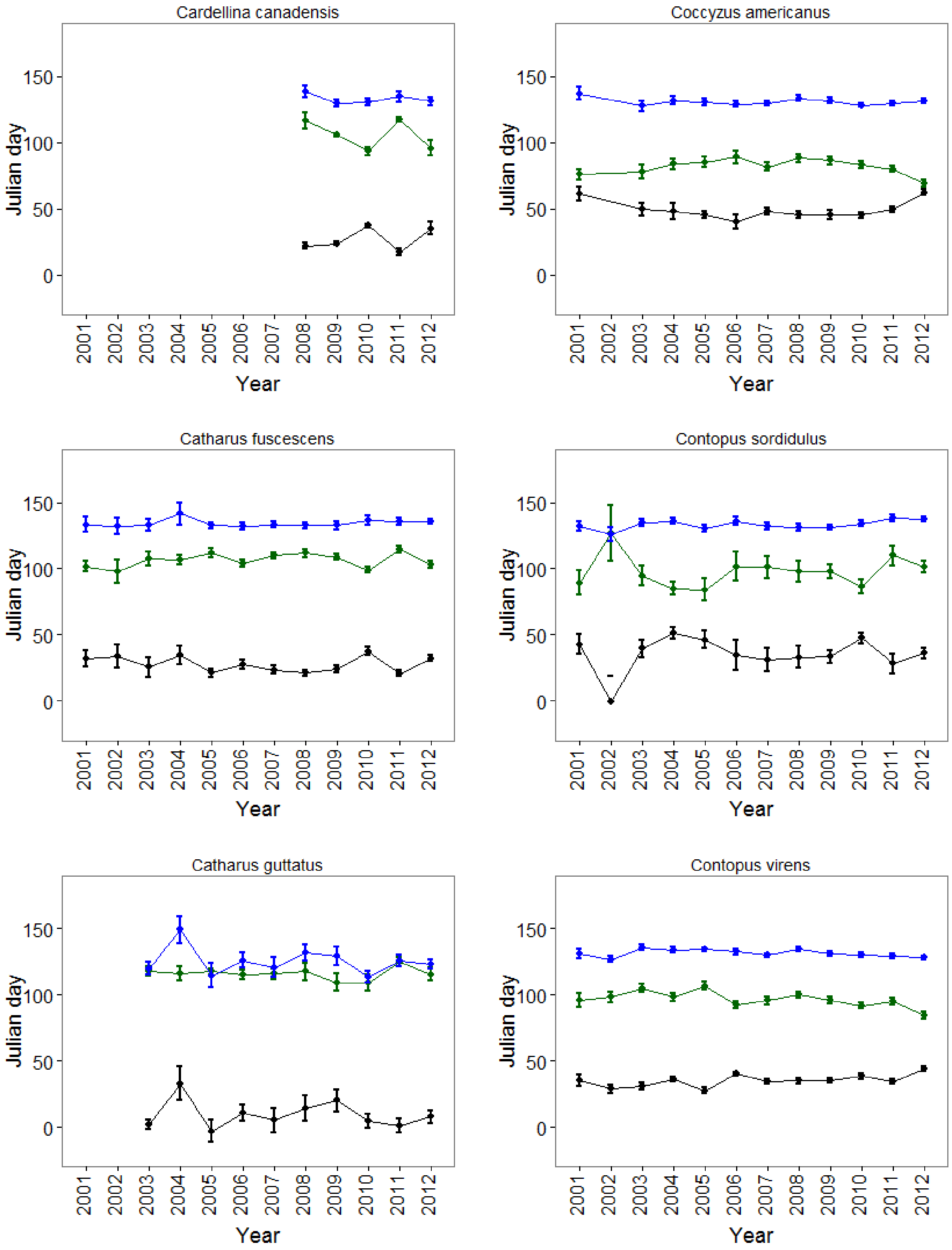

Cardellina canadensis
Coccyzus americanus
Catharus fuscescens
Contopus sordidulus
Catharus guttatus
Contopus virens
Julian day
150
100
50
0
2001
2002
2003
2004
2005
2006
2007
2008
2009
2010
2011
2012
Year

Supplementary Figure S5. Trajectories of green-up (green), arrival (blue), and phenological interval (black) for each species. Bars indicate standard error of mean estimated over all grid cells.

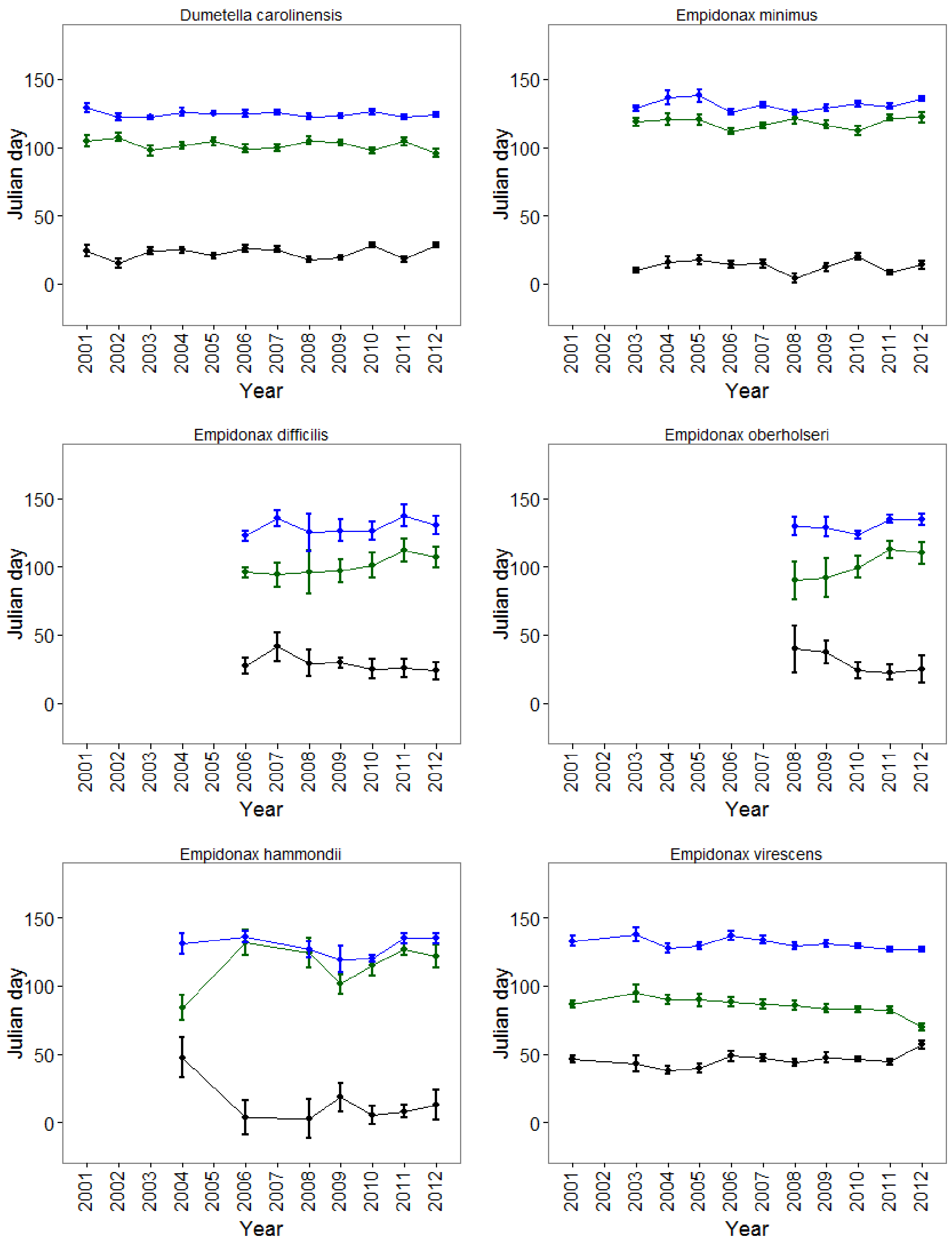

Supplementary Figure S5, continued.

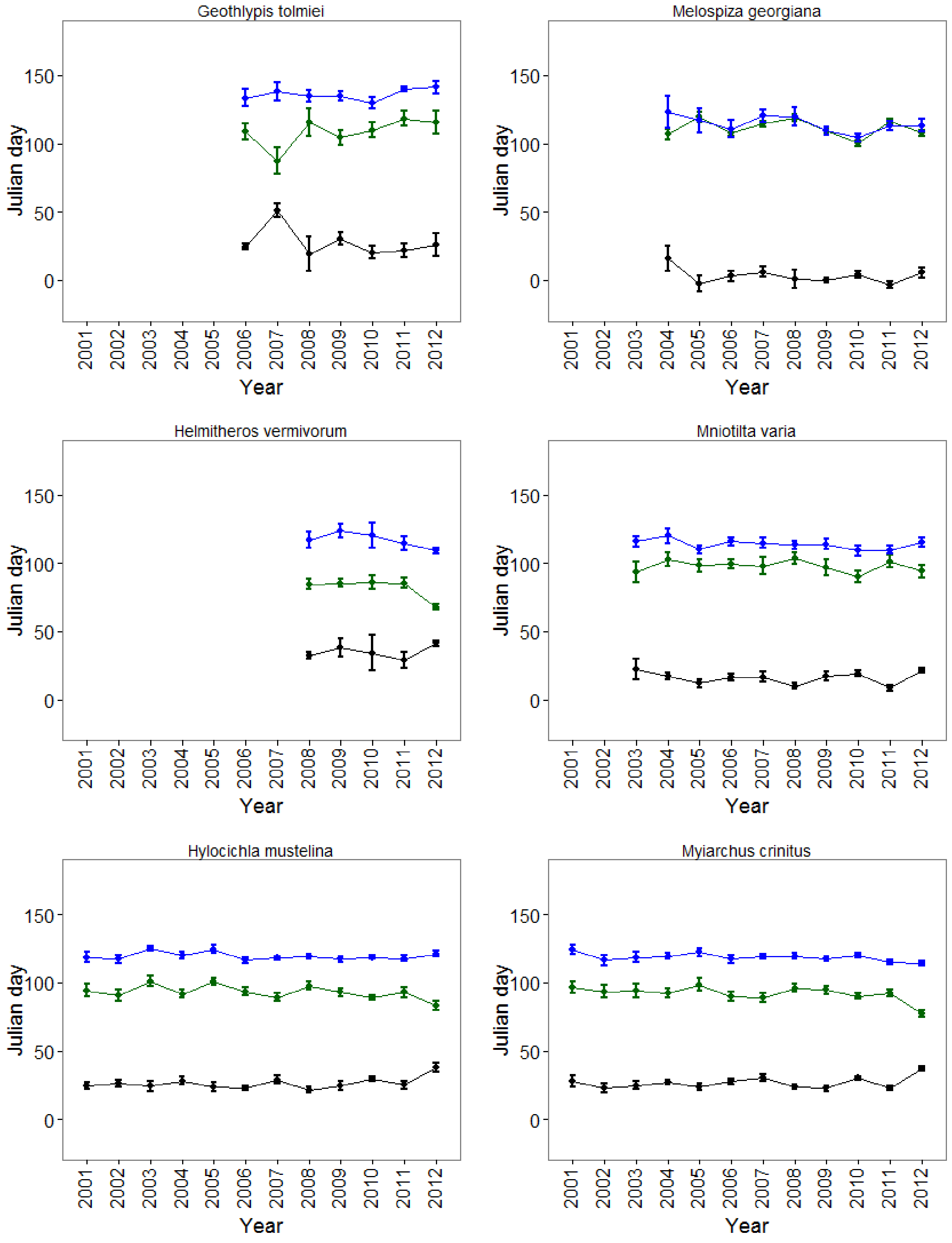

Supplementary Figure S5, continued.

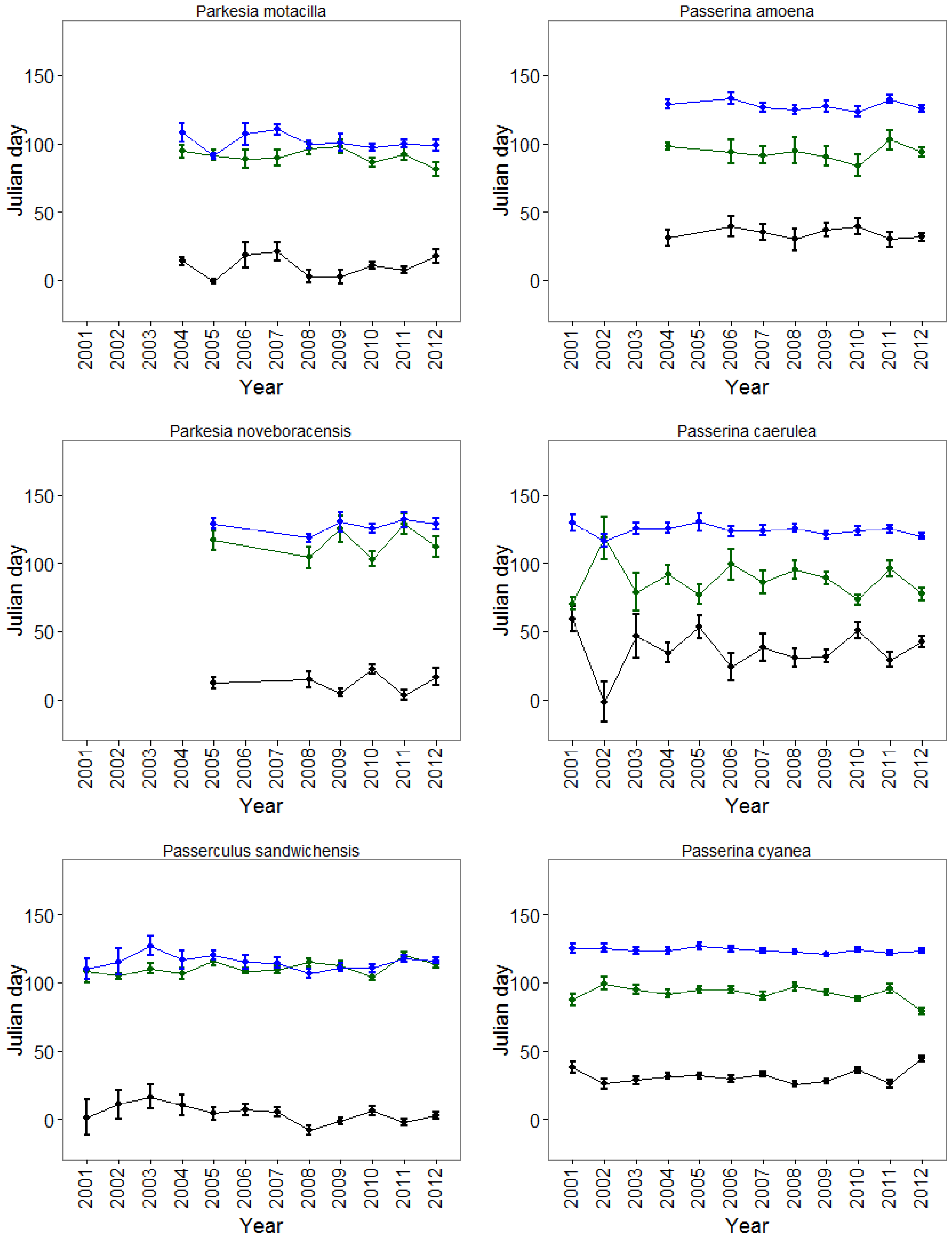

Supplementary Figure S5, continued.

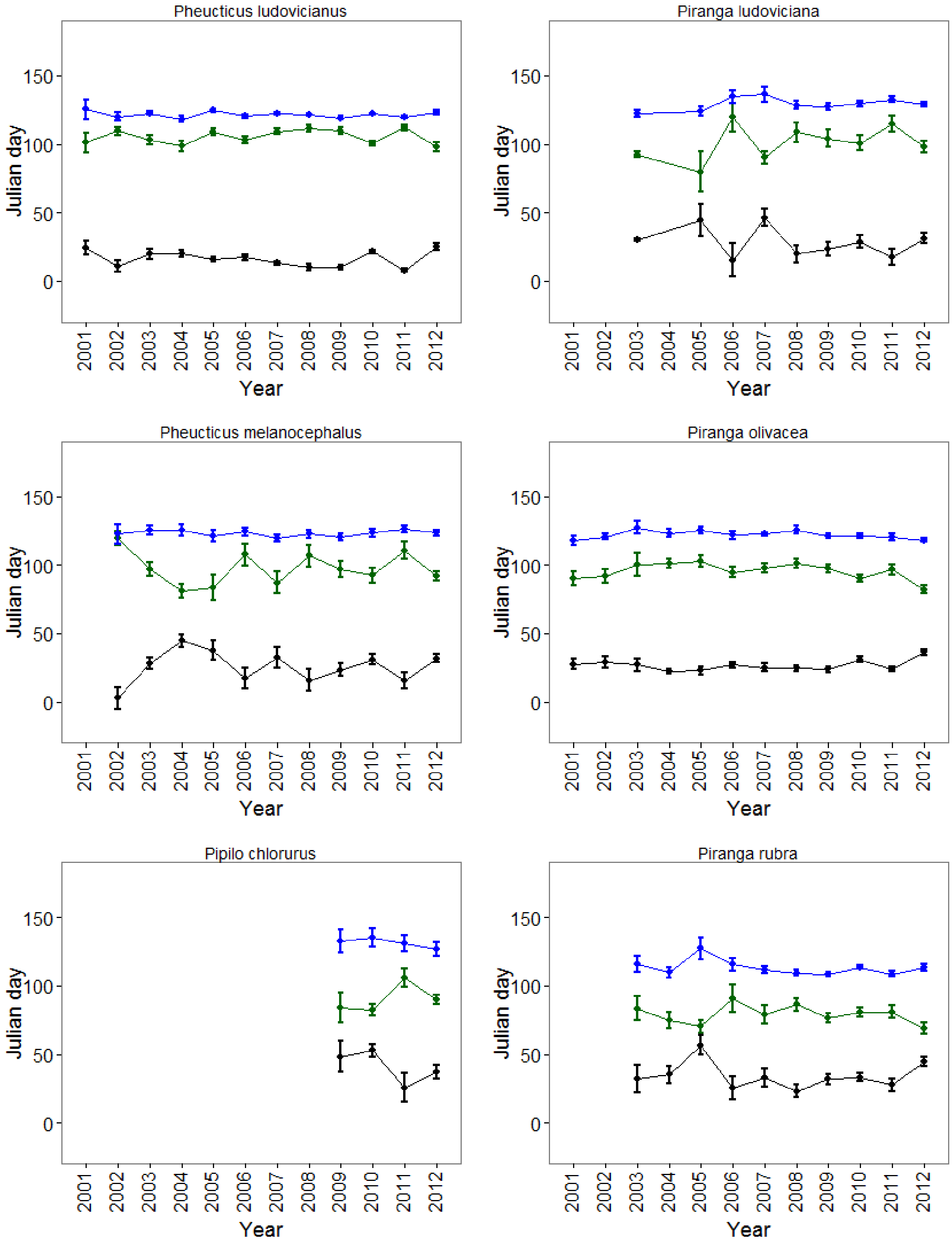

Supplementary Figure S5, continued.

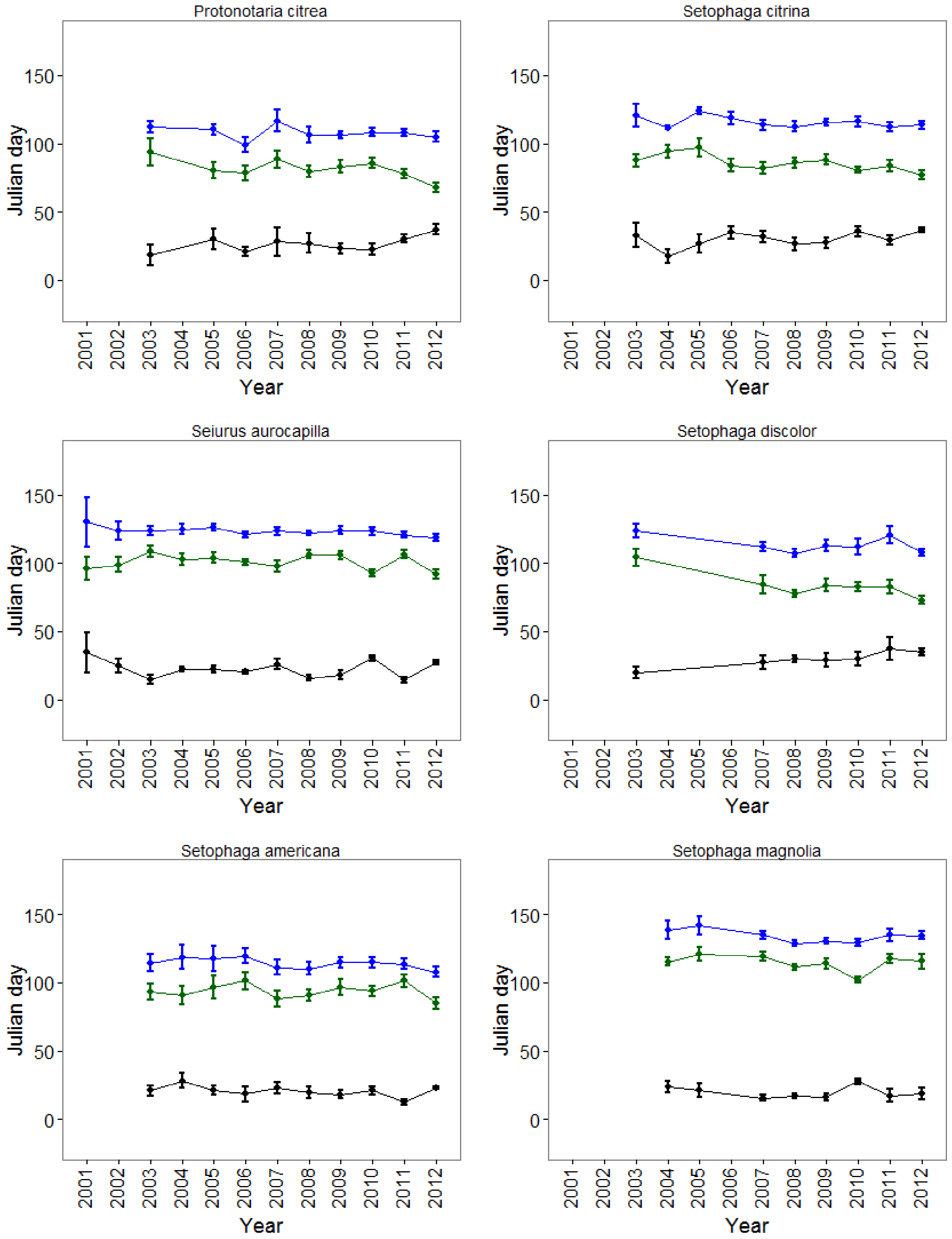

Supplementary Figure S5, continued.

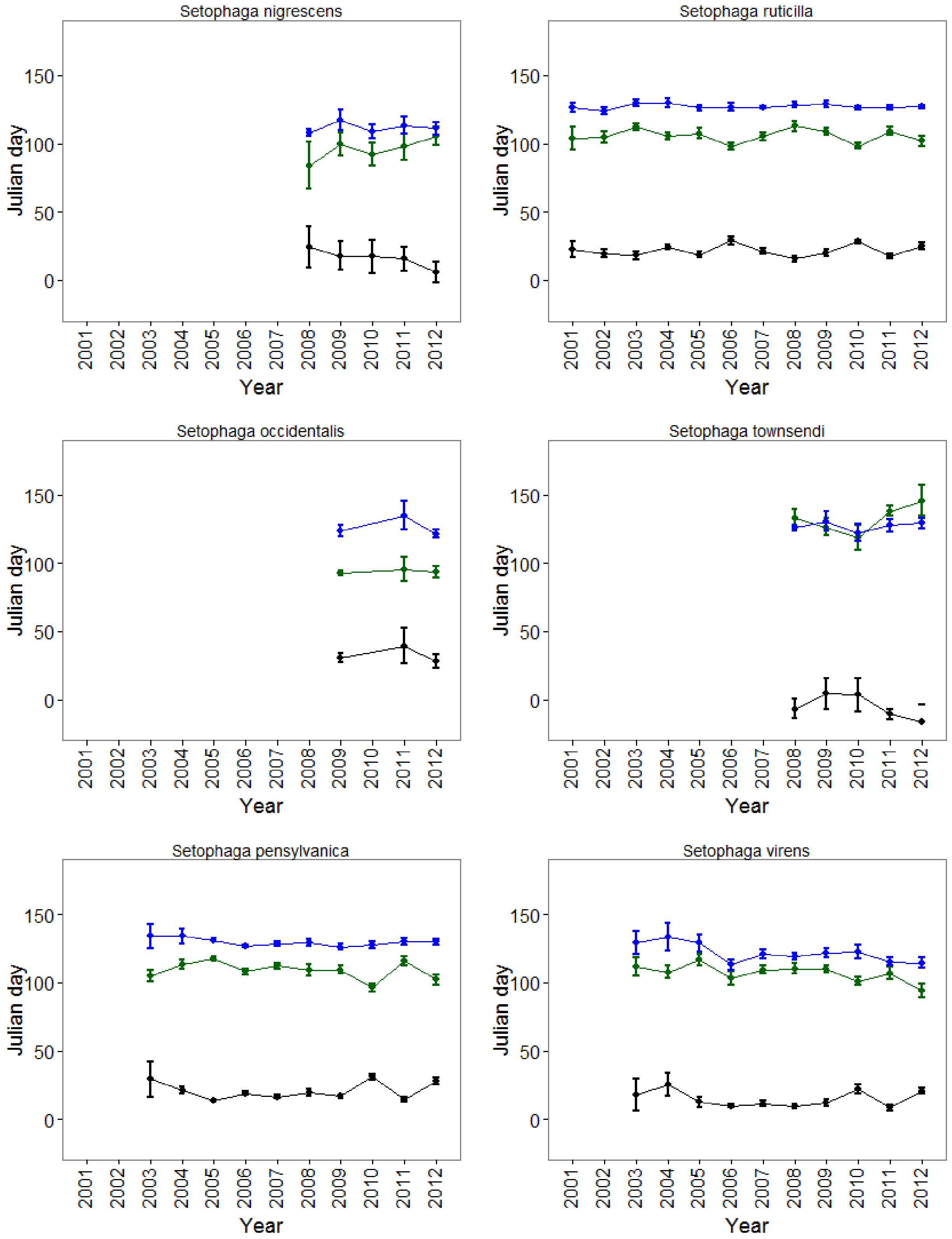

Supplementary Figure S5, continued.

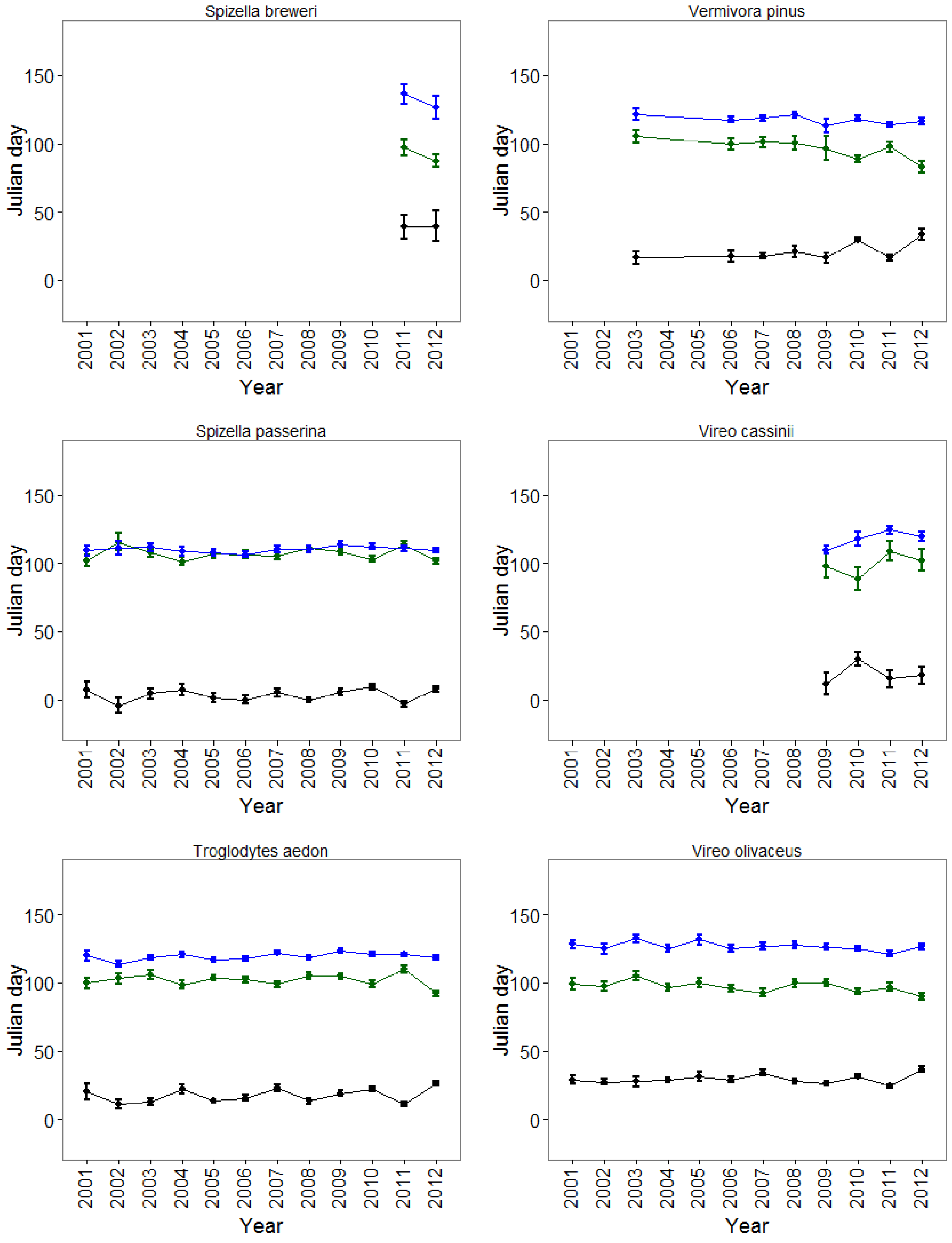

Supplementary Figure S5, continued.

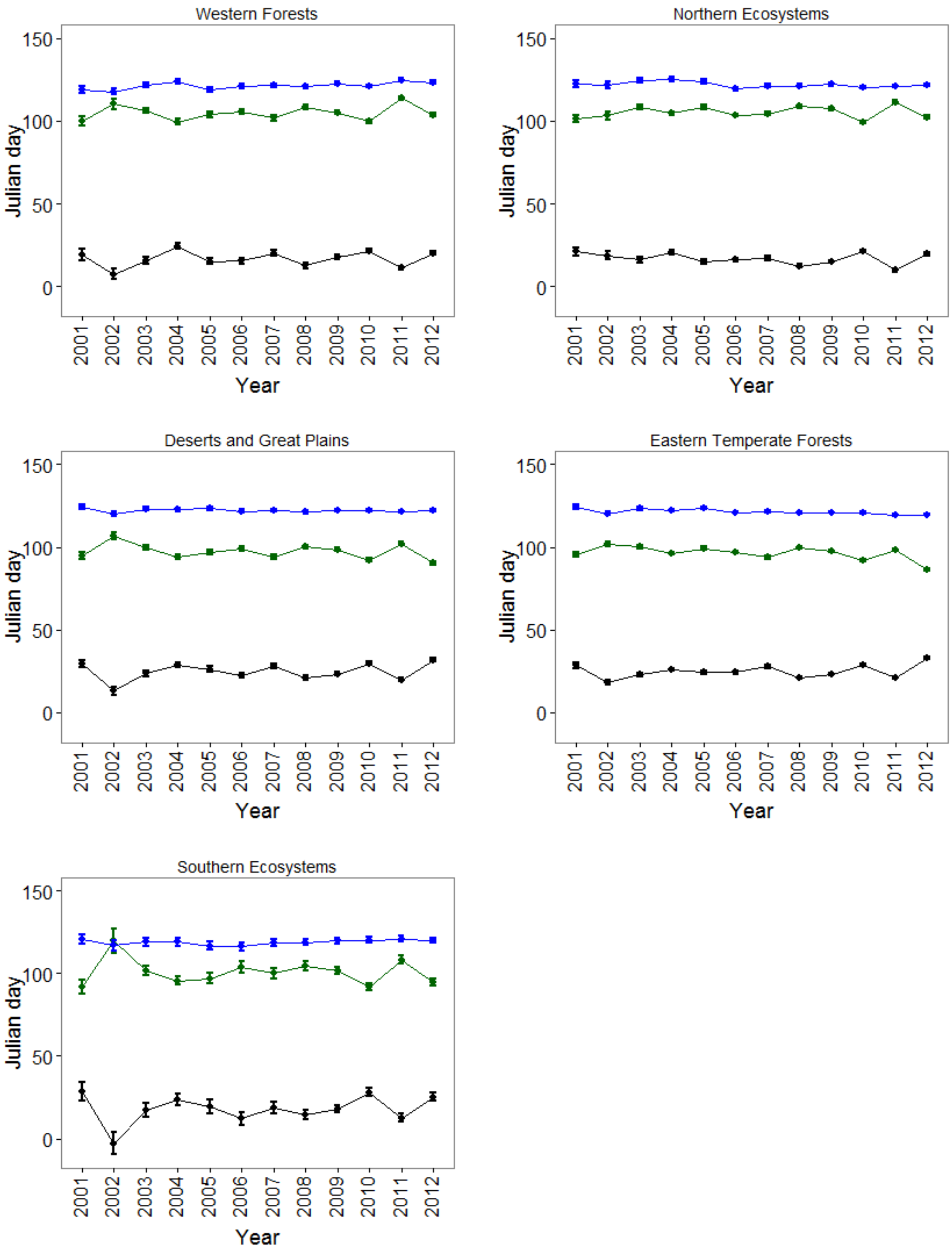

Supplementary Figure S6. Trajectories of green-up (green), arrival (blue), and phenological interval (black) within each ecoregion. Bars indicate standard error. Ecoregions as in Supplementary Fig. S2.

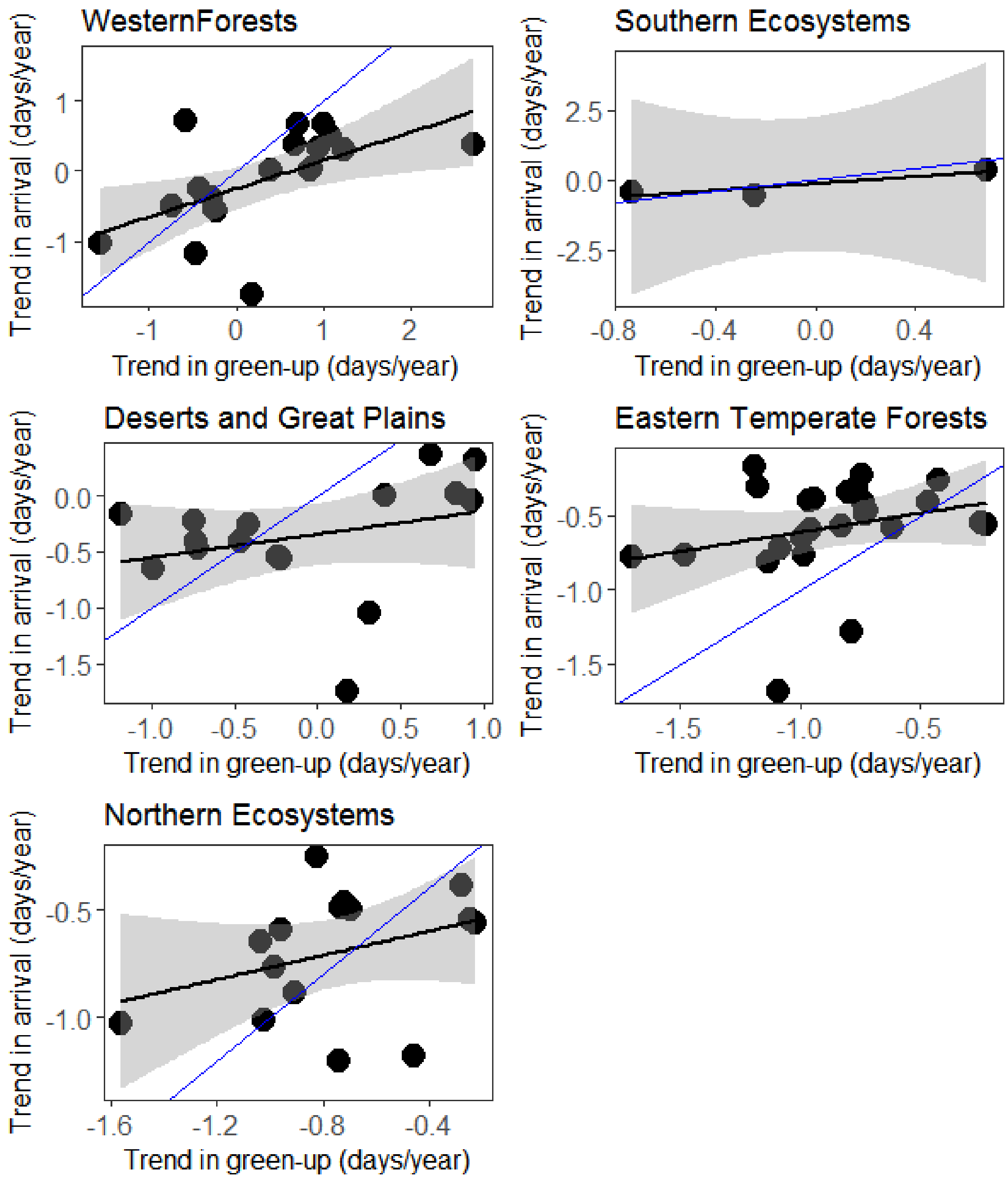

Supplementary Figure S7. Relationship between trend in bird species arrival and trend in green-up for each ecoregion. Each symbol represents a species. Black line indicates linear regression for all species, blue line indicates where trend in arrival equals trend in green-up. Grey regions indicate 95% confidence bands. Ecoregions as in Supplementary Fig. S2.

Supplementary Table S1. Green-up trends as estimated by linear mixed models. Models estimated for cells of a species' breeding range. Asterisks indicate significance at $p < 0.05$.

Species	Intercept	Slope	SE	*p*	
Cardellina canadensis	2157.725	-1.021	0.576	0.088	
Catharus fuscescens	1510.078	-0.697	0.181	<0.001	*
Catharus guttatus	1029.725	-0.454	0.301	0.136	
Coccyzus americanus	2468.563	-1.188	0.250	<0.001	*
Contopus sordidulus	-1268.405	0.683	0.510	0.182	
Contopus virens	1987.929	-0.943	0.121	<0.001	*
Dumetella carolinensis	1032.710	-0.463	0.122	<0.001	*
Empidonax difficilis	-1326.643	0.713	0.590	0.232	
Empidonax hammondii	-2387.238	1.246	0.431	0.006	*
Empidonax minimus	681.754	-0.279	0.217	0.201	
Empidonax oberholseri	-1808.814	0.951	0.696	0.182	
Empidonax virescens	2351.003	-1.130	0.178	<0.001	*
Geothlypis tolmiei	-1914.216	1.008	0.479	0.042	*
Helmitheros vermivorum	2269.628	-1.090	0.454	0.026	*
Hylocichla mustelina	1756.148	-0.829	0.133	<0.001	*
Melospiza georgiana	1598.565	-0.740	0.290	0.013	*
Mniotilta varia	2017.466	-0.955	0.232	<0.001	*
Myiarchus crinitus	2016.289	-0.960	0.124	<0.001	*
Parkesia motacilla	1325.485	-0.617	0.329	0.066	
Parkesia noveboracensis	3257.272	-1.561	0.466	0.003	*
Passerculus sandwichensis	572.381	-0.226	0.189	0.233	
Passerina amoena	-724.169	0.406	0.476	0.396	
Passerina caerulea	1560.626	-0.734	0.653	0.262	
Passerina cyanea	1584.302	-0.744	0.126	<0.001	*
Pheucticus ludovicianus	1636.595	-0.762	0.177	<0.001	*
Pheucticus melanocephalus	-1594.761	0.843	0.492	0.088	
Pipilo chlorurus	-538.048	0.314	0.544	0.568	
Piranga ludoviciana	-1638.410	0.867	0.647	0.183	
Piranga olivacea	1706.015	-0.802	0.149	<0.001	*
Piranga rubra	2072.559	-0.992	0.483	0.042	*
Protonotaria citrea	3492.772	-1.699	0.344	<0.001	*
Seiurus aurocapilla	2076.781	-0.983	0.147	<0.001	*
Setophaga americana	1667.954	-0.785	0.229	0.001	*
Setophaga citrina	2261.608	-1.086	0.222	<0.001	*
Setophaga discolor	3049.794	-1.478	0.328	<0.001	*
Setophaga magnolia	1768.076	-0.823	0.301	0.009	*
Setophaga nigrescens	-1775.462	0.931	0.968	0.344	
Setophaga occidentalis	1248.452	-0.574	2.126	0.790	
Setophaga pensylvanica	2183.786	-1.032	0.245	<0.001	*

Setophaga ruticilla	1573.549	-0.731	0.137	<0.001	*
Setophaga townsendi	-5362.070	2.730	0.807	0.003	*
Setophaga virens	1926.231	-0.906	0.229	<0.001	*
Spizella breweri	-274.051	0.182	0.674	0.791	
Spizella passerina	596.499	-0.243	0.156	0.119	
Troglodytes aedon	944.280	-0.420	0.117	<0.001	*
Vermivora pinus	2451.547	-1.173	0.381	0.003	*
Vireo cassinii	-2070.041	1.077	0.437	0.019	*
Vireo olivaceus	1546.906	-0.722	0.111	<0.001	*
	Mean	-0.372	0.387		
	Min	-1.699			
	Max	2.730			

Supplementary Table S2. Arrival trends as estimated by linear mixed models. Asterisks indicate significance at $p < 0.05$.

Species	Intercept	Slope	SE	*p*	
Cardellina canadensis	2170.582	-1.014	0.556	0.079	
Catharus fuscescens	1135.069	-0.497	0.241	0.041	*
Catharus guttatus	2490.920	-1.177	0.557	0.037	*
Coccyzus americanus	463.868	-0.166	0.195	0.397	
Contopus sordidulus	-606.264	0.370	0.164	0.025	*
Contopus virens	913.025	-0.390	0.106	<0.001	*
Dumetella carolinensis	951.345	-0.411	0.076	<0.001	*
Empidonax difficilis	-1188.921	0.656	0.636	0.306	
Empidonax hammondii	-464.232	0.296	0.584	0.615	
Empidonax minimus	909.214	-0.386	0.183	0.038	*
Empidonax oberholseri	-506.474	0.317	0.577	0.585	
Empidonax virescens	1757.086	-0.810	0.217	<0.001	*
Geothlypis tolmiei	-1205.101	0.668	0.556	0.235	
Helmitheros vermivorum	3507.298	-1.687	0.624	0.013	*
Hylocichla mustelina	1254.587	-0.565	0.129	<0.001	*
Melospiza georgiana	2521.912	-1.198	0.553	0.033	*
Mniotilta varia	1309.329	-0.595	0.208	0.005	*
Myiarchus crinitus	922.189	-0.401	0.087	<0.001	*
Parkesia motacilla	1266.284	-0.580	0.303	0.060	
Parkesia noveboracensis	2187.244	-1.024	0.476	0.041	*
Passerculus sandwichensis	1245.124	-0.562	0.305	0.066	
Passerina amoena	114.801	0.006	0.240	0.980	
Passerina caerulea	956.315	-0.414	0.294	0.161	
Passerina cyanea	573.056	-0.224	0.110	0.041	*
Pheucticus ludovicianus	732.766	-0.304	0.071	<0.001	*
Pheucticus melanocephalus	104.610	0.011	0.147	0.943	
Pipilo chlorurus	2251.747	-1.056	0.621	0.097	
Piranga ludoviciana	74.668	0.028	0.225	0.900	
Piranga olivacea	794.188	-0.335	0.126	0.008	*
Piranga rubra	1431.938	-0.656	0.280	0.020	*
Protonotaria citrea	1659.901	-0.772	0.409	0.063	
Seiurus aurocapilla	1664.186	-0.767	0.211	<0.001	*
Setophaga americana	2689.005	-1.282	0.290	<0.001	*
Setophaga citrina	1555.593	-0.718	0.305	0.021	*
Setophaga discolor	1650.475	-0.765	0.459	0.101	
Setophaga magnolia	645.542	-0.255	0.352	0.472	
Setophaga nigrescens	186.733	-0.037	0.581	0.949	
Setophaga occidentalis	-1315.029	0.716	0.894	0.437	
Setophaga pensylvanica	1439.916	-0.652	0.218	0.004	*

Setophaga ruticilla	1116.850	-0.492	0.101	<0.001	*
Setophaga townsendi	-642.705	0.384	0.600	0.528	
Setophaga virens	1893.169	-0.882	0.287	0.003	*
Spizella breweri	3648.815	-1.750	1.083	0.123	
Spizella passerina	1206.100	-0.544	0.127	<0.001	*
Troglodytes aedon	646.345	-0.262	0.120	0.030	*
Vermivora pinus	736.357	-0.308	0.186	0.105	
Vireo cassinii	-867.729	0.489	0.537	0.368	
Vireo olivaceus	1057.720	-0.464	0.118	<0.001	*
	Mean	-0.426	0.340		
	Min	-1.750			
	Max	0.716			

Supplementary Table S3. Phenological interval trends as estimated by linear mixed models. Asterisks indicate significance at $p < 0.05$.

Species	Intercept	Slope	SE	*p*	
Cardellina canadensis	-430.909	0.228	0.729	0.756	
Catharus fuscescens	-178.761	0.103	0.298	0.731	
Catharus guttatus	2101.648	-1.041	0.609	0.091	
Coccyzus americanus	-2032.621	1.036	0.316	0.001	*
Contopus sordidulus	625.316	-0.295	0.516	0.568	
Contopus virens	-1110.887	0.572	0.141	<0.001	*
Dumetella carolinensis	-104.145	0.064	0.135	0.637	
Empidonax difficilis	535.352	-0.254	0.791	0.749	
Empidonax hammondii	2529.310	-1.252	0.733	0.095	
Empidonax minimus	413.532	-0.200	0.274	0.468	
Empidonax oberholseri	1333.793	-0.649	0.842	0.447	
Empidonax virescens	-753.011	0.399	0.255	0.120	
Geothlypis tolmiei	1358.237	-0.662	0.753	0.383	
Helmitheros vermivorum	999.199	-0.479	0.739	0.523	
Hylocichla mustelina	-451.387	0.239	0.173	0.169	
Melospiza georgiana	533.573	-0.265	0.513	0.607	
Mniotilta varia	-348.557	0.182	0.287	0.529	
Myiarchus crinitus	-1112.927	0.569	0.149	<0.001	*
Parkesia motacilla	-28.934	0.021	0.438	0.961	
Parkesia noveboracensis	-196.194	0.103	0.738	0.889	
Passerculus sandwichensis	1310.217	-0.652	0.342	0.058	
Passerina amoena	838.663	-0.400	0.530	0.453	
Passerina caerulea	-476.640	0.256	0.715	0.720	
Passerina cyanea	-1006.504	0.517	0.173	0.003	*
Pheucticus ludovicianus	-844.565	0.428	0.201	0.034	*
Pheucticus melanocephalus	1410.590	-0.689	0.520	0.187	
Pipilo chlorurus	2618.494	-1.285	0.888	0.156	
Piranga ludoviciana	1523.986	-0.745	0.649	0.253	
Piranga olivacea	-998.852	0.511	0.172	0.003	*
Piranga rubra	-784.598	0.407	0.535	0.448	
Protonotaria citrea	-2006.535	1.014	0.515	0.053	
Seiurus aurocapilla	-297.487	0.159	0.247	0.521	
Setophaga americana	1477.051	-0.725	0.347	0.039	*
Setophaga citrina	-820.498	0.424	0.375	0.261	
Setophaga discolor	-1559.456	0.793	0.503	0.120	
Setophaga magnolia	-606.428	0.311	0.426	0.467	
Setophaga nigrescens	1909.227	-0.942	0.924	0.316	
Setophaga occidentalis	-2513.576	1.265	2.461	0.615	
Setophaga pensylvanica	-732.910	0.375	0.300	0.215	

Setophaga ruticilla	-441.012	0.230	0.169	0.173	
Setophaga townsendi	5013.580	-2.493	0.972	0.018	*
Setophaga virens	-382.613	0.198	0.352	0.575	
Spizella breweri	3953.118	-1.947	1.383	0.174	
Spizella passerina	509.524	-0.251	0.189	0.184	
Troglodytes aedon	-251.590	0.135	0.156	0.387	
Vermivora pinus	-1540.151	0.778	0.384	0.047	*
Vireo cassinii	1576.816	-0.775	0.583	0.193	
Vireo olivaceus	-526.427	0.277	0.147	0.060	
	Mean	-0.092	0.512		
	Mean absolute effect size	0.575			
	Min	-2.493			
	Max	1.265			

Supplementary Table S4. Adjustment of significance tests for trends in phenological interval considering multiple comparisons. Species are ordered by raw p values. Raw *p* values indicate no adjustment for multiple comparisons, with significance 'a' given when raw *p* < *α* and *α* = 0.05. The Bonferroni test controls the familywise error rate (Dunn, 1961). Significance 'b' is given when the raw *p* value is less than the Bonferroni critical value *a* / *m* , where α = 0.05 and *m*, the number of tests = 48. The Benjamin-Hochberg (BH) test controls the false discovery rate (Benjamini & Hochberg, 1995). Significance 'c' and 'd' are given for false discovery rates (FDR) of 0.05 and 0.25 respectively when the raw *p* value is less than its BH critical value (*i/m*)*Q* where *i* is the rank of the raw *p*, and *Q* is the FDR selected. The cutoffs required for significance of the raw *p* value were: for a familywise error rate of 0.05, 0.001042; for a FDR of 0.05, 0.003377; and for a FDR of 0.25, 0.059748.

Species	Raw *p*	Bonferroni critical value	BH critical value when FDR = 0.05	BH critical value when FDR = 0.25	Signifi-cance
Contopus virens	0.000060	0.001042	0.001042	0.005208	a, b, c, d
Myiarchus crinitus	0.000153	0.001042	0.002083	0.010417	a, b, c, d
Coccyzus americanus	0.001160	0.001042	0.003125	0.015625	a, c, d
Passerina cyanea	0.003007	0.001042	0.004167	0.020833	a, c, d
Piranga olivacea	0.003377	0.001042	0.005208	0.026042	a, c, d
Setophaga townsendi	0.018275	0.001042	0.006250	0.031250	a, d
Pheucticus ludovicianus	0.034253	0.001042	0.007292	0.036458	a, d
Setophaga americana	0.038955	0.001042	0.008333	0.041667	a, d
Vermivora pinus	0.047308	0.001042	0.009375	0.046875	a, d
Protonotaria citrea	0.052629	0.001042	0.010417	0.052083	d
Passerculus sandwichensis	0.058255	0.001042	0.011458	0.057292	d
Vireo olivaceus	0.059748	0.001042	0.012500	0.062500	d
Catharus guttatus	0.090815	0.001042	0.013542	0.067708	
Empidonax hammondii	0.095377	0.001042	0.014583	0.072917	
Empidonax virescens	0.120240	0.001042	0.015625	0.078125	
Setophaga discolor	0.120306	0.001042	0.016667	0.083333	
Pipilo chlorurus	0.155917	0.001042	0.017708	0.088542	
Hylocichla mustelina	0.169216	0.001042	0.018750	0.093750	
Setophaga ruticilla	0.173125	0.001042	0.019792	0.098958	
Spizella breweri	0.174330	0.001042	0.020833	0.104167	
Spizella passerina	0.184448	0.001042	0.021875	0.109375	
Pheucticus melanocephalus	0.186639	0.001042	0.022917	0.114583	
Vireo cassinii	0.192709	0.001042	0.023958	0.119792	
Setophaga pensylvanica	0.214519	0.001042	0.025000	0.125000	
Piranga ludoviciana	0.253062	0.001042	0.026042	0.130208	
Setophaga citrina	0.261110	0.001042	0.027083	0.135417	
Setophaga nigrescens	0.316317	0.001042	0.028125	0.140625	
Geothlypis tolmiei	0.383497	0.001042	0.029167	0.145833	

Troglodytes aedon	0.386852	0.001042	0.030208	0.151042
Empidonax oberholseri	0.446590	0.001042	0.031250	0.156250
Piranga rubra	0.448059	0.001042	0.032292	0.161458
Passerina amoena	0.452508	0.001042	0.033333	0.166667
Setophaga magnolia	0.467314	0.001042	0.034375	0.171875
Empidonax minimus	0.467785	0.001042	0.035417	0.177083
Seiurus aurocapilla	0.520976	0.001042	0.036458	0.182292
Helmitheros vermivorum	0.523356	0.001042	0.037500	0.187500
Mniotilta varia	0.528967	0.001042	0.038542	0.192708
Contopus sordidulus	0.567872	0.001042	0.039583	0.197917
Setophaga virens	0.575139	0.001042	0.040625	0.203125
Melospiza georgiana	0.607159	0.001042	0.041667	0.208333
Setophaga occidentalis	0.614962	0.001042	0.042708	0.213542
Dumetella carolinensis	0.636719	0.001042	0.043750	0.218750
Passerina caerulea	0.720432	0.001042	0.044792	0.223958
Catharus fuscescens	0.731247	0.001042	0.045833	0.229167
Empidonax difficilis	0.749457	0.001042	0.046875	0.234375
Cardellina canadensis	0.756014	0.001042	0.047917	0.239583
Parkesia noveboracensis	0.889272	0.001042	0.048958	0.244792
Parkesia motacilla	0.961086	0.001042	0.050000	0.250000

References

Benjamini Y, Hochberg Y (1995) Controlling the False Discovery Rate: A Practical and Powerful Approach to Multiple Testing. *Journal of the Royal Statistical Society B*, **57**, 289–300.

Dunn O (1961) Multiple Comparisons Among Means. Journal of the American Statistical Association **56**, 52–64.

Supplementary Note S1. Analysis of effects of passing migrants on arrival date estimates

Observations of migratory birds may consist of two groups of individuals. Individuals who arrive and remain for the breeding season will be termed “breeders”, while individuals who arrive in a cell but quickly pass through and depart to another cell will be termed “passers”. Our interest is primarily in breeders as these individuals may be more reliably associated with broad environmental conditions and their synchrony with the environment may be more important for demography. However, we were unable to differentiate among breeders and passers. Here we offer several evaluations of the impact of this limitation on our results, an important step in leveraging the exceptional richness of eBird or similar data to facilitate our understanding of phenological mismatch.

There are several possible reasons why passers may be less of a problem than one may at first imagine. First, phenological interval between arrival and environment may occur and be relevant for both passers and breeders, even if the case for demographic impacts of high phenological intervals is greater for breeders. Second, passers will only bias studies of phenological interval if they arrive at substantially different times than individuals remaining to breed. Third, phenological interval trends were more strongly driven by green-up than by arrival (Fig. 1).

Although we could not exclude passers from the analysis, we investigated the potential sensitivity of our results to passers in three ways. First, passers are expected to decrease with latitude within a species’ range, so we re-estimated phenological interval trends while controlling for latitude (see note 1a). We found that more species showed significant trends in phenological interval when controlling for latitude, indicating both the sensitivity to latitude and that our estimates of the number of species with significant phenological interval trends may have been conservative. Second, if passers influence the proportion of presences observed over time (for example, by arriving later than breeders), then the estimation of arrival dates may be sensitive to the range in dates (window size) over which logistic curves were estimated. This effect may vary with latitude if passers are influential. We investigated this sensitivity and found that arrival was sensitive to window size but that this effect did not vary with latitude (see note 1b). Third, if passers arrive later than breeders, our estimates of arrival dates may be biased late. We re-estimated the arrival dates at several points on the logistic curves to simulate earlier arrival and found that more species displayed significantly increasing phenological interval trends and that the mean phenological interval trend was stronger, indicating that our estimates may have been conservative (see note 1c).

1a. Controlling for latitudinal effect on phenological interval.

We expected that the proportion of passers of any given species would decrease with latitude. Individuals on the northernmost edge of a breeding range would, for example, likely consist only of breeders. Although exceptions may exist to this pattern, it may serve as a general assumption to test the impacts of passers on phenological interval trends. If this assumption is valid, and if passers decrease our ability to detect phenological interval trends by biasing arrival date estimation, we expected that controlling for latitude would reduce the number of species for which significant trends in phenological interval would be observed.

To test if latitude was a strong predictor of phenological interval, we modeled phenological interval as follows, implemented with package lme4 and lmerTest in R:

Phenological interval = *Latitude* + *Year** + *Cell** + *Species**

where * denotes random intercept, and *Year* was fit as a categorical factor.

We found latitude was a strong predictor of phenological interval, as shown in the following table.

	Estimate	SE	*t*	*p*
Intercept	46.03	6.82	6.75	< 0.001
Latitude	-0.564	0.16	-3.61	< 0.001

Several factors may account for the impact of latitude on phenological interval, including: fewer passage migrants at higher latitudes (within a species), or increased fitness pressure on phenological synchrony with green-up in more seasonal environments typically at higher latitudes.

We re-modelled trends in phenological interval for each species using the method described in the main text, except that we controlled for latitude with the following model specification:

Phenological interval = *Latitude* + *Year** + *Cell**

where * denotes random intercept.

We found that when controlling for latitude, phenological interval increased significantly in 35 of 48 species (73%). This contrasts our finding that when not controlling for latitude (see main text), phenological interval increased significantly in 9 of 48 species (19%).

We conclude that if latitude is a good proxy for the proportion of passers, passers are unlikely to have resulted in “false positives”, that is, falsely identifying increasing phenological interval in species. Our results suggest phenological interval trends may be more widespread across species than our analyses ignoring latitude revealed.

1b. Sensitivity analysis of logistic curve window size on arrival dates.

It is important that the logistic regression models employed appropriately estimate mean arrival dates of bird populations. However, logistic curve fits can be impacted by the behavior of occurrences following the arrival of a population. For example, if following their arrival to a cell, a large proportion of migrants continue north to other cells, these passers may generate a downturn in the proportion of occurrences that are presences.

We tested the impact of temporal window size on arrival date by refitting logistic curves for all cell-species-years at for windows of Julian days 80-160, 80-180, and 80-200. We then fit a linear mixed model using R's 'lme4' package:

Arrival date = window size + year + species + cell*,

where * denotes random intercept.

We determined that estimated arrival date is significantly impacted by window size ($p < 0.001$).

However, what is important is not whether this effect exists, but whether this effect of window size on arrival date varies systematically with geography, because that could bias our geographically based results. We therefore tested for the impact of latitude, longitude, and elevation on the effect of window size on arrival date. To do so, we first determined the effect of the window size on arrival date for each individual cell. We then fit a linear regression (window on arrival date effect = centroid latitude + centroid longitude + mean elevation) with cell as the sample unit.

We determined that the effect of window size on arrival date was independent of latitude, longitude, and elevation, as shown in the table below.

	Estimate	SE	*t*	*p*
Intercept	0.00324	0.04836	0.067	0.947
Latitude	0.00007	0.00101	0.068	0.946
Longitude	0.00024	0.00044	0.557	0.578
Elevation	<0.00001	0.00001	0.025	0.980

Next we tested if the effect of window size on arrival date varies by ecoregion, using the same procedure as above. We applied a Bonferroni correction to the p-values to account for multiple comparisons. The results, as shown in the table below, indicate that effect of window size on arrival date does not significantly relate to ecoregion

	Estimate	SE	*t*	*p*
Intercept	0.05629	0.06247	0.901	1.00
Northern Ecosystems	-0.05679	0.06271	-0.906	1.00
Eastern Temperate Forests	-0.08054	0.06271	-1.284	1.00
Deserts & Great Plains	0.01016	0.05171	0.196	1.00
Western Forests	-0.05259	0.02237	-2.351	0.121
Southern Ecosystems	-0.03647	0.06609	-0.552	1.00

These results mirror those of Hurlbert & Liang (2012, PLoS One 7:e31662), who in a similar analysis altered the window sizes relative to original arrival date estimates and found that although window size marginally impacted arrival date, this impact did not vary significantly with latitude or migration distance.

1c. Sensitivity analysis of arrival date estimation on phenological interval trends.

The estimated arrival dates used in the main study indicate the inflection point of the logistic curve fit to the proportion of presences over time. They represent the mean arrival date of the population to the given cell by a given species in a given year, and correspond to the date at which 0.5 of the asymptotic (maximum) proportion of presences was reached.

However, if passers both a) make up a substantial proportion of observed individuals in a species, and b) systematically arrive earlier or later than breeders, then our estimates of arrival may be biased. For example, if passers tend to arrive later than breeders, our estimated arrival dates may be biased late. Passers may be more likely to arrive later than breeders because earlier arriving migrants often breed at lower latitudes (Conklin *et al.*, 2010) and early arrival to a breeding location can be beneficial to fitness (Smith & Moore, 2005).

To determine the sensitivity of phenological interval trends to arrival date estimation, we re-estimated arrival dates. Using the same fitted logistic regression curves, we determined the dates on the curves when *x* proportion of the asymptotic proportion of presences was reached.

Where the original model fit in R was:

nls (*PropP* ~ (*Asym* / (1 + exp ((*xmid* – *Julian*) / *scal*)))

and where *PropP* is the proportion of presences, *Asym* is the asymptote, *xmid* is the date at which 0.5 of the asymptote is reached, *julian* is the date of year, and *scal* is the scale parameter.

we set *PropP* = *x*(*Asym*), where *x* is the proportion of the asymptotic proportion of presences, here *x* was set as 0.25, 0.333, and 0.666.

used the parameters fit by the model, and solved for *julian* as follows:

$julian_x = xmid - (scal * (\log ((Asym / PropP) – 1)))$.

We then re-calculated phenological interval with these new arrival dates, and re-estimated trends in phenological interval over time, all using the same method as described in the main text.

We found that the number of species for which significantly increasing phenological interval ($\alpha = 0.05$) was observed decreased with the proportion of the asymptotic proportion of presences (see table below).

Arrival dates	*x* proportion of asymptote	No. spp. with increasing phenological interval		
		Positive	Negative	Total
Earlier	0.25	16	3	19
	0.333	13	3	16
	0.5	7	2	9
Later	0.666	5	2	7

We also found that the strength of the phenological interval trend increased with the proportion of the asymptotic proportion of presences (see table below). All models fit as in main study according to:

abs ($Async_x$) ~ *Year* + *Species** + *Cell**

where * denotes random intercept.

Arrival dates	*x* proportion of asymptote	Absolute phenological interval trend (days/year)*	SE	*t*	*p*
Earlier	0.25	0.272	0.038	7.02	< 0.001
	0.333	0.219	0.040	5.49	< 0.001
	0.5	0.115	0.044	2.65	0.008
Later	0.666	0.001	0.005	0.214	0.830

*Based on absolute value of phenological interval

These observations suggest that if arrival dates were influenced by passers, then the number of species with significantly increasing phenological interval, and the overall strength in phenological interval trend, were likely sensitive to the proportion of passers in the data and the discrepancy in arrival dates between passers and breeders. The results also suggest that if passers arrived later than breeders such that earlier arrival dates are more representative of breeders, then we may have underestimated the number of species with increasing phenological interval trends and the strength of phenological interval trends. If instead passers arrived earlier than breeders such that later arrival dates are more representative of breeders, we may have overestimated the number of species with increasing phenological interval trends and the strength of phenological interval trends.

Since if passers and breeders arrive at different times, passers are likely to arrive later than breeders, we conclude that our estimates of the number of species and of the strength of phenological interval trends in the main study (where $x = 0.5$) are likely conservative.

References

Benjamini Y, Hochberg Y (1995) Controlling the False Discovery Rate : A Practical and Powerful Approach to Multiple Testing. *Journal of the Royal Statistical Society B*, **57**, 289–300.

Conklin JR, Battley PF, Potter MA, Fox JW (2010) Breeding latitude drives individual schedules in a trans-hemispheric migrant bird. *Nature communications*, **1**, 67.

Dunn O (1961) Multiple Comparisons Among Means. **56**, 52–64.

Kelling S, Johnston A, Hochachka WM et al. (2015) Can observation skills of citizen scientists be estimated using species accumulation curves? *PLoS ONE*, **10**, 1–20.

Kosmala M, Wiggins A, Swanson A, Simmons B (2016) Assessing data quality in citizen science. *Frontiers in Ecology and the Environment*, **14**, 551–560.

Smith RJ, Moore FR (2005) Arrival timing and seasonal reproductive performance in a long-distance migratory landbird. *Behavioral Ecology and Sociobiology*, **57**, 231–239.

Sullivan BL, Aycrigg JL, Barry JH et al. (2014) The eBird enterprise: An integrated approach to development and application of citizen science. *Biological Conservation*, **169**, 31–40.

Supplementary Note S2: Citizen science data quality

Citizen science data used in this study was obtained from eBird and like any data collected by many different observers, these checklists can vary in quality (Sullivan *et al.*, 2014; Kelling *et al.*, 2015; Kosmala *et al.*, 2016). For instance, Kelling et al. (2015) showed that more species are reported in checklists by observers who have reported a greater number of checklists. If data quality varied systematically across space or time, our results could have been impacted, even after considering that we included only those arrival date estimates from models with good fit and statistical significance (see Methods). We therefore analyzed this data (Table 2 in Kelling et al., 2015) to test whether the mean number of checklists reported by the lowest and highest quartiles of eBird observers varied by Bird Conservation Region (BCR). We performed a general linear model in R as follows:

Checklists = Quartile + BCR

where Quartiles and BCR were categorical variables.

We found that the number of checklists did not vary significantly by region:

	Degrees of freedom	Sum of squares	Mean squared	*F*	*p*
Quartile	1	18404	18404	19.902	0.007
BCR	5	5080	1016	1.098	0.460
Residuals	5	4625	925		

We conclude that regional variation in the number of checklists reported per observer was not significant and that regional variation in data quality was unlikely to impact our primary results.

Refernces:

Kelling S, Johnston A, Hochachka WM et al. (2015) Can observation skills of citizen scientists be estimated using species accumulation curves? *PLoS ONE*, **10**, 1–20.

Kosmala M, Wiggins A, Swanson A, Simmons B (2016) Assessing data quality in citizen science. *Frontiers in Ecology and the Environment*, **14**, 551–560.

Sullivan BL, Aycrigg JL, Barry JH et al. (2014) The eBird enterprise: An integrated approach to development and application of citizen science. *Biological Conservation*, **169**, 31–40.
